# Metaphors of young-onset dementia in the illness narratives of those with the condition

**DOI:** 10.1371/journal.pone.0314717

**Published:** 2024-12-03

**Authors:** Flaminia Miyamasu

**Affiliations:** Medical English Communications Center, Institute of Medicine, University of Tsukuba, Tsukuba, Ibaraki, Japan; University of Valencia, SPAIN

## Abstract

This study identified and analyzed metaphors related to the lived experience of young-onset dementia that were used in nine illness narratives written by people with the condition. A final set of 1111 MEs sorted into 30 source domain categories were grouped according to six target domain categories reflecting the biologic (the person with dementia’s body/brain), psychologic (suffering with dementia, coping with dementia, dementia itself, the person with dementia), and social (the social experience of dementia) aspects of having dementia. Notably, many of the metaphors were similar to previously reported metaphors of illness, such as fight and journey, and other metaphors of embodiment, as well as disease as enemy, body as container, and body as machine. In addition, although negative conceptualizations were in the majority, almost one-third of the metaphoric expressions, belonging to the fight and journey source domain categories, reflected mainly positive images. The commonality of metaphor types with those of other illness experiences supports the notion of shared metaphors across illness contexts. Moreover, in contrast to the dehumanizing and stigmatizing terms that have previously been used to socially construct dementia, the positivity of metaphoric images identified here indicates the authors’ proactive and affirming conceptualizations of their experience of dementia. Health care professionals can draw on this study’s findings to help their own patients make sense of and cope with dementia.

## Introduction

Dementia is a major neurocognitive disorder caused by various disease processes including Alzheimer disease, cerebrovascular disease, and frontotemporal lobar disease [1]. These disease processes cause progressive declines in memory, reasoning, use of language, and task execution, among others, thereby significantly curtailing the affected individual’s social, occupational, and intellectual functioning [1]. Symptoms can appear before the age of 65 years, in which case the condition is called young-onset (or early-onset) dementia; in the United Kingdom, young-onset dementia (YOD) accounts for 7.5% of those with dementia [2]. Although life-style changes and treatments can slow the progression of dementia, a cure is currently unavailable [1], and in several countries surveyed, dementia has been shown to be the second-most feared disease, after cancer [3]. Given the condition’s progressive nature, it is important to understand how people with dementia perceive their condition while their cognitive functioning remains relatively intact as such information might be used to inform care later on when the ability to articulate experience and needs may be less intact [4]. The metaphoric language used by people with YOD, for example, might shed light on the phenomenal world of those with the condition.

### Conceptual metaphor

According to Conceptual Metaphor Theory (CMT) [5, 6], one of the ways in which we make sense of experience is through conceptual metaphor (CM), when we think about one conceptual domain of experience in terms of another. This transfer of meaning, or cross-domain mapping, is typically from a more concrete conceptual domain, the source domain, to a more abstract domain, the target domain, and results in more concrete understanding of the target domain. The CM manifests at the level of language as metaphoric expressions (MEs). To illustrate, anger is fire is a CM [6] in which the more concrete components of the source domain fire (combustion, heat, red color, and so forth) map onto the more abstract components of the target domain anger. Examples of MEs that make this CM manifest at the level of language are:

She was *fuming*.

He made *inflammatory* remarks.

That made my blood *boil*.

His rage was *white hot*.

(Note that CMs are conventionally formatted with small capitals, and MEs, with italics [7]). The systematic structuring of the concept of anger through these different but semantically related MEs renders a coherent view of the anger target domain, thereby making it more concrete in our understanding [6]. As the first three of these examples show, the target domain is often implicit (or else mentioned earlier) [8].

Metaphoric expressions are pervasive in everyday English language use; indeed, they may have become so conventionalized that we use them without being aware of their metaphoricity [9]. Having become thus conventionalized, MEs would be listed in a dictionary, but usually after the more basic, ie, nonmetaphoric, sense of the word [9]. For example, taking one of the aforementioned MEs, the first sense given in the Oxford Dictionary of English [10] of the intransitive verb “to fume” is “emit gas, smoke, or vapour,” whereas the second sense given is its conventional metaphoric one of “feel, show, or express great anger.” In contrast to conventional metaphors, novel metaphors are typically not listed in a dictionary [9]; examples of novel metaphors are “pilgrim soul” and “life is a box of chocolates.”

### Metaphor studies of illness

Since CM facilitates more concrete understanding of abstract conceptual domains, one of its functions is to express aspects of experience that may be otherwise difficult to express [11]. Illness is one such experience that may be “beyond words” [12], and metaphors have provided rich ground for researchers to examine the illness experience, for example, studies on metaphors of pain [13], cancer [8, 14–17], depression [18, 19], voice-hearing [20], post-traumatic stress disorder [21], and motor neuron disease (MND) [22]. The findings from these studies revealed typical metaphors to describe illness, such as fight/war [8, 14, 15, 17, 18, 20, 22] and journey [8, 14–18, 22], with implications for better understanding of patients’ conceptual worlds and preferred communication styles [8, 12–17, 19, 20, 22].

### Metaphor studies of dementia

Dementia metaphor studies have fallen into roughly two types: (1) those examining metaphors used at the societal level to describe dementia and (2) those examining metaphors used by people directly or indirectly affected by the condition—patients, caregivers, and health care professionals. Examples of the former type of study include the study by Behuniak [23], who argued that the presence of the zombie metaphor in both the scholarly literature (eg, biomedical articles) and the popular literature (eg, movies, novels) has contributed to a stigmatizing and dehumanizing social construction of people with dementia. In a similar vein, Zeilig [24] examined how metaphoric representations of dementia across a range of narratives in the political, medico-scientific, and sociocultural spheres have shaped the collective understanding of dementia as something negative and fearful. The publication of Zimmermann’s paper [25], in 2017, brought a bridging between the study of societally used metaphors and those used by people actually affected by dementia, as the author examined the metaphors used in the autobiographical accounts of those with the condition or of their caregivers and how these metaphors both mirror the social discourse around dementia (eg, metaphors of war, mind as broken machine) and counter it (eg, metaphors of dementia as an enriching experience, such as journey).

Examples of studies focusing exclusively on metaphors used by those directly or indirectly affected by the condition include that by Golden and colleagues [26], in which the six most frequent core metaphors to emerge from interviews with spouses of people with dementia were journey, machine/circuit, up/down, harm/abuse, game, and hand; on the basis of these findings, the authors offered practical suggestions on how nurses might apply these metaphors to aid family caregivers’ understanding and well-being. Two other studies focused on the metaphoric representation of dementia in blog narratives. The first of these [27] was of three blogs written by Flemish authors: two family caregivers of people with dementia and one health professional. The findings revealed meaningful differences in the metaphor uses of the caregivers and the health care professional: whereas the caregivers focused more on their personal experiences of living with a person with dementia, the health care professional was concerned more with the behavioral problems of people with dementia and with appropriate communication strategies. The second study [28] was of blogs written exclusively by people with dementia. Narrowing down the study target to metaphors related to well-being in dementia, the author identified 12 metaphor groups (the four most frequent: loss, war/combat, robbery, journey) and discussed the implications of the study’s findings for patient-centered care.

### Aim of this study

Whilst the findings of these previous metaphor studies on the lived experience of dementia have provided valuable insights into the condition, the studies’ focus on caregivers’ experience, blog narratives, or a particular aspect of the person with dementia’s experience means that gaps in the research remain. Towards filling those gaps, I here conducted a systematic analysis of the most frequently used metaphors related to the experience of YOD in a selection of illness narratives written by those with the condition in the hope that the findings might contribute to a broader understanding of the experience and needs of people with YOD. Specifically, the study sought to answer the following research questions: (1) What CMs do the authors of these illness narratives use to conceptualize their experience of YOD? (2) How do these CMs align with those identified in previous metaphor studies of dementia and of other illnesses?

## Methods

### Illness narratives

The illness narratives analyzed here were obtained by first conducting a Google search using various combinations of the keywords “dementia,” “young-onset dementia,” “early-onset dementia,” “narratives,” “memoirs,” and “autobiographies.” This initial search led to a web page on the Dementia UK website dedicated to the topic of autobiographies of people with young-onset dementia [29], which in turn led to an Amazon search for similar sorts of autobiographies. The illness narratives thus obtained were then narrowed down and selected according to the following criteria: written in English and authors identified themselves as having dementia that was diagnosed when they were aged younger than 65 years. A balance of male- and female-authored illness narratives was selected. For ease of metaphor extraction, where possible, the e-book (Kindle) format was used. Since the illness narratives are published and freely available in the public domain, institutional ethical approval was not sought.

### Metaphor analysis

An inductive approach was taken; in other words, no a priori assumptions were made as to the types or patterns of metaphors that would emerge during the course of the study. A systematic analysis of all the MEs in the corpus related to the lived experience of dementia was carried out according to the six-step procedure described below.

1. *Broad-based identification of candidate MEs* Each illness narrative was annotated line by line for candidate MEs (conventional and novel) related to the lived experience of dementia.
2. *Identification and extraction of MEs for analysis* The metaphoricity of each candidate ME was verified using a modified version (ie, using steps 3b and 4) of the well-established Metaphor Identification Procedure of the Pragglejaz Group [30]. Briefly, a lexical unit (ie, the smallest unit of meaning in a sentence, eg, a word, noun phrase, or phrasal verb [31]) was verified as metaphoric when it was judged to have a more basic meaning than that being used in the context but to be still understandable in comparison with the basic meaning. For example, in the expression “this *journey* with dementia,” the basic sense of the word is evidently not being used; instead, correspondences between the physical act of taking a journey, in which various events and encounters may be experienced, are being made with the experience of having and living with dementia; thus, the sense of “journey” here is verified as metaphoric. All words and phrases thus verified as metaphoric were extracted from the text along with the immediate co-text (including the target domain if explicitly stated) and pasted into spreadsheets.
3. *Sorting of MEs by source domain and labeling of resultant categories* The MEs were sorted into categories according to semantically related source domain terms, and the categories, labeled accordingly [32]. Thus, “this *journey* with dementia” was grouped with MEs such as “those who are *on their own path* with this disease,” and the source domain category was labeled being on a journey.
4. *Formation of CMs by grouping of source domain categories according to shared target domain* Next, the source domain categories were grouped according to the target domain they were judged to be implicitly or explicitly describing. For example, the target domain for being on a journey was judged to be “coping with dementia,” and the combination of target domain and source domain labels led to the formation of the CM coping with dementia is being on a journey.
5. *Quantification of MEs and formation of the final metaphor dataset for analysis* The number of MEs per source domain category and the distribution of each within the dataset were calculated. Then, to ensure that the MEs to be analyzed were representative of the illness narratives as a whole, source domain categories not containing MEs from a majority of the authors (ie, five of the nine illness narratives) were discarded, to leave a final dataset of MEs for qualitative analysis.
6. *Assessment of labeling trustworthiness* To ensure labeling accuracy and consistency, metaphor groupings and labels were independently verified by two other analysts. Interrater reliability was assessed using Miles and Huberman’s formula: Reliability = Number of agreements / Total number of agreements + Disagreements, in which an interrater agreement score higher than 90% is taken to reflect interrater reliability [33].

## Results

### Illness narratives

Nine dementia illness narratives that fit the inclusion criteria were selected, at a ratio of 5:4 male:female authors. The narratives were published between 1993 and 2021; two were coauthored. The authors’ average age at diagnosis was 55 years (range, 45–64 years). Table 1 shows the illness narrative titles and relevant authors’ details.

**Table 1:** Illness narrative titles and authors’ details.

Illness narrative title	Author’s sex	Age at diagnosis, y	Dementia diagnosis
*Living in the labyrinth*: *A personal journey through the maze of Alzheimer’s* (LIL)^a^ [34]	F	45	Early-onset Alzheimer disease
*Losing my mind*: *An intimate look at life with Alzheimer’s* (LMM) [35]	M	57	Early-onset Alzheimer disease
*Dancing with dementia*: *My story of living positively with dementia* (DWD) [36]	F	46	Alzheimer disease, later rediagnosed as frontotemporal dementia
*Finding my way*, *losing myself*: *A short memoir of early onset Alzheimer’s dementia* (FMW) [37]^b^	M	52	Early-onset Alzheimer disease
*Memory’s last breath*: *Field notes on my dementia* (MLB) [38]	F	60	Cerebral microvascular disease (precursor of vascular dementia)
*On Pluto*: *Inside the mind of Alzheimer’s* (OP) [39]	M	59	Early-onset Alzheimer disease
*Dear Alzheimer’s*: *A diary of living with dementia* (DA) [40]	M	54	Young-onset Alzheimer disease
*Somebody I used to know* (UTK) [41]	F	58	Young-onset dementia
*A tattoo on my brain*: *A neurologist’s personal battle against Alzheimer’s disease* (TAT) [42]^b^	M	64	Early-stage Alzheimer disease

^**a**^Acronyms following narrative titles are those used in Table 3 and S1 Table.

^**b**^Coauthored with one other person

### Quantitative results

**Fig 1:**
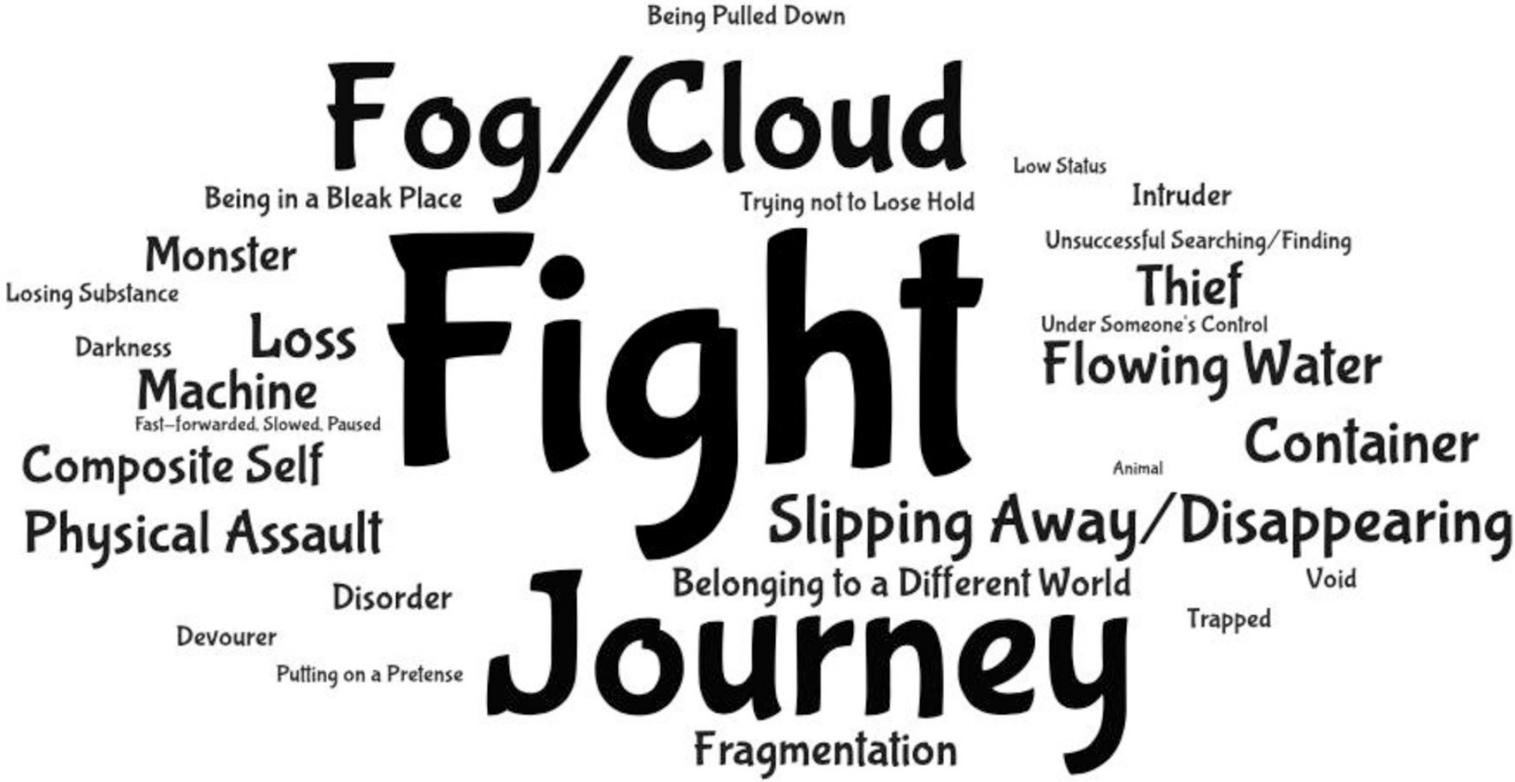
Word cloud of source domain categories identified in this study. (Generated using WordArt.com, https://wordart.com).

Initially, 1521 MEs related to the authors’ lived experiences of dementia were extracted and sorted into 65 source domain categories. Elimination of source domain categories not containing MEs from at least five of the illness narratives left a final dataset for analysis of 1111 MEs sorted into 30 source domain categories. These source domain categories were further grouped according to six emergent target domain categories: suffering with dementia, coping with dementia, dementia itself, the person with dementia’s body/brain, the person with dementia, and the social experience of dementia, which reflected the biologic (the person with dementia’s body/brain), psychologic (suffering with dementia, coping with dementia, dementia itself, the person with dementia), and social (the social experience of dementia) aspects of the lived experience of dementia. The verification procedure conducted by two independent analysts resulted in the relabeling of one of the source domain categories; otherwise, there was broad agreement with how the source and target domains were grouped and labeled (interrater reliability: 98.4%). The most noteworthy quantitative finding was that whilst negative conceptualizations were in the majority, almost one-third (29.3%) of the MEs belonged to the coping with dementia target domain category, which represented mostly positive conceptualizations of living with dementia. Table 2 shows the distribution of MEs from each target domain category, and the word cloud in Fig 1, the source domain categories.

**Table 2:** Target domain categories and distribution of MEs.

Target domain category	MEs, n	%
suffering with dementia	442	39.8
coping with dementia	326	29.3
dementia itself	114	10.3
the person with dementia’s body/brain	98	8.8
the person with dementia	69	6.2
the social experience of dementia	62	5.6
Total	1111	

### Qualitative results

The most noteworthy qualitative finding was the emergence of CMs that have been previously identified as conventional metaphors of illness: illness as fight [8, 14, 15, 17, 18, 20, 22], illness as journey [8, 14–18, 22], and other metaphors of embodiment [8, 22], as well as disease as enemy [43], body as container [5], and body as machine [43]. Table 3 provides representative MEs for the CMs, which are described in detail below. The S1 Table provides the complete final dataset of MEs analyzed, along with quantitative information.

**Table 3:** Conceptual metaphors with representative metaphorical expressions (MEs).

Target domain	Source domain, with representative ME
suffering with dementia is …	being in fog/cloud
	“wait for *the fog to lift*. How did it *descend* so quickly? Like driving on a bright day right into *a thick cloud*” (UTK^**a**^165^**b**^)
	being at the mercy of flowing water
	“I will *float on a tranquil sea of memory* one moment and be *swept away* the next by *boisterous waves* that leave me confused and uncertain” (LMM42)
	enduring physical assault
	“what I am enduring. It’s like *water torture*: drip, drip, drip” (FMW872)
	being in a state of disorder
	“mind *jumbled* in *a junkyard piled with* anxiety” (LMM167)
	being in a void
	“We feel as if we are hanging onto a high cliff, above a lurking *black hole*” (DWD98)
	being in darkness
	“That’s Alzheimer’s. A *light goes off*, and one goes into a rage because it’s *dark*” (OP317-8)
	being trapped
	“I was mentally *mired in quicksand*” (LIL88)
	being pulled down
	“the powerful pull of the Alzheimer’s *downward spiraling trajectory* never let up” (OP312)
	trying not to lose hold
	“for Marie’s personable letters. They helped me *hang on* when I felt there was *nothing to hang onto*” (LIL75)
	being under someone else’s control
	“before long it will have *complete and absolute control over* you” (LMM108)
	being fast-forwarded, slowed down, or paused
	“… early-onset Alzheimer’s; it’s a death *in slow motion*. *A freeze frame* at times” (OP7)
	things slipping away/disappearing
	“my more recent memories and thoughts *slip away* from me like a helium balloon” (FMW678)
	loss
	“the square I know so well but which had been *lost* in an instant” (UTK166)
	fragmentation
	“Life has become *a fragmented kaleidoscope* of problems” (DWD98)
	unsuccessful searching/finding
	“Words… before they were *retrievable* and now *they are not*” (LMM31)
coping with dementia is …	being in a fight
	(war)
	“I don’t want to just sit here and wait for this disease *to make its march on* my mind” (UTK103)
	“These fairly simple choices become *an organized counterattack against* Alzheimer’s” (TAT125)
	(race/chase)
	“I had *to sprint*, a full-out panic *dash*, to avoid capture at sundown” (OP203)
	“I will do everything I can *to keep one step ahead* of you” (DA4084)
	(general)
	“I will have to wipe the frown off my face and *show my teeth* more often” (LMM86)
	“You get *knocked down*, you get back up. Again and again. You find a way to *win*” (OP6)
	“you’ve *won some rounds* but I still feel like I’m still *winning the bout*” (DA2747)
	being on a journey
	“We devote our time, attention, and energies to *the road we are on*, not *the destination*” (LIL138)
	Like the Pluto spacecraft itself, we’re all on *a journey* to unknown places” (OP202)
	“My *journey w*ith dementia has been *a journey* of self-discovery about who I really am” (DWD158)
	“I believe that people with dementia are making *an important journey* from cognition, through emotion, into spirit” (DWD158)
dementia itself is …	a thief
	“a *mind-stealing* illness” (LMM151)
	a monster
	“this *demon* prowling like Abaddon” (OP8)
	an intruder
	“as the disease *claims more mental territory*” (LMM76)
	a devourer
	“[dementia] *feasts on* Wernicke’s area… *gorges itself on* Broca’s area” (MLB66)
the person with dementia’s body/brain is …	a container
	“an increasingly flawed *filing system* of my brain” (DA236)
	a machine
	“My brain, on this day, is not responding. The *rainbow icon* is spinning again” (OP187)
the person with dementia is …	a composite self
	“I do wake up and wonder, *Which me* am I today?” (UTK134)
	a person who’s losing substance
	“I write memoir… to flesh out *my shrinking self* with former selves” (MLB44)
	a person of low status
	“I doubted his efficiency in that regard, but crossed my fingers. *Beggars* cannot be choosers” (LIL90)
	an animal
	“I *rattle my cage* but no one comes *to feed* me” (LMM185)
the social experience of dementia is …	belonging to a different world
	“It is as if we are *bi-cultural*, and have stepped across *the divide between your world and ours*” (DWD170)
	being in a bleak place
	“remarkable parallels between Alzheimer’s and *Pluto*: dense isolation, penetrating silence, a harsh environment, and a world of unthinkable contrasts” (OP4)
	putting on a pretense
	“Life had become *an improvisational theater*” (LIL64)

^**a**^For full forms of acronyms, see Table 1.

^**b**^Numbers following acronyms indicate page numbers or e-book locations.

### Suffering with dementia CMs

This, the largest target domain category, consisted of 15 CMs comprising 442 MEs. The CMs could in turn be grouped according to whether the cause of suffering was conceptualized as arising from outside or from within the person. Source domain categories conceptualizing the cause of suffering as arising from outside the person were being in fog/cloud; being at the mercy of flowing water; enduring physical assault; being in a state of disorder; being in a void; being in darkness; being trapped; being pulled down; trying not to lose hold; being under someone else’s control; and being fast forwarded, slowed down, or paused. These metaphors relate to the body in interaction with the outside world. Since embodied experience is fundamental to how human beings make sense of the world, embodied metaphors are common, including to describe the illness experience. Indeed, Gibbs and Franks [8] pointed out that the majority of the metaphors used by their study participants to describe cancer were based in everyday, healthy embodied experiences. What is noteworthy about the embodied metaphors of dementia found here is that rather than being based in healthy embodied experiences, they are based in some sort of threatened embodied experience, frequently one due to a natural element or force—weather, water, physical force, physical surroundings, motion. These conceptualizations of threatened embodied experience highlight the person with dementia’s sense of vulnerability, powerlessness, and fear.

Source domain categories conceptualizing the cause of suffering as arising from within the person were things slipping away/disappearing, loss, fragmentation, and unsuccessful searching/finding. These metaphors relate to a state of progressive attrition and reflect the person with dementia’s sense of progressive personal loss, with progressive impairments in cognition conceptualized as things disintegrating and gradually slipping away before being lost altogether.

### Coping with dementia CMs

This target domain category, the second-largest, consisted of two source domain categories: being in a fight and being on a journey. Containing the largest number of MEs of this study (212), the being in a fight source domain category was further divided into three subcategories according to the type of fighting evoked: war, race/chase, and general. The fight metaphor describes difficult experiences and lends itself particularly well to the illness experience through its close cross-domain mapping: enemy–disease, combatant–patient, commander–doctor, weaponry–treatments, military allies–health care team [17]. Taken from this stance, the fight metaphor is an empowering one, offering as it does a proactive conceptualization of aggressive action-taking rather than of passivity in the face of difficulty [14, 15, 17]. From another stance, however, the metaphor can have disempowering aspects [14, 15, 17, 43], for example, the conceptualization also inherent in the metaphor of failure to survive corresponding to failure to fight hard enough [17]. Whilst both empowering and disempowering fight metaphors were present in the current dataset, the fact that this was the most numerous of all the MEs used by these authors with YOD indicates their tenacity and will not to give up even in the face of a far-from-guaranteed victorious outcome. Indeed, the very act of writing, witnessing to the experience of dementia through the telling of their narratives, may be a part of the authors’ “fight” against oncoming dependency, decline, and passivity [25].

Like the fight metaphor, the journey metaphor has its empowering and its disempowering aspects: the journey may proceed smoothly, the patient/traveler may be in charge of the journey and have a traveling companion(s), or he/she may not [14, 15]. Notably, whilst the metaphor in the current findings was framed in both empowering and disempowering ways, the journey was invariably described as a relentlessly forward-moving one. In other words, the journey of dementia conceptualized here does not fit a typical illness narrative type in which the journey is presented as a quest from which the person who has experienced illness returns with some boon or wisdom learned [44]. Nor is the dementia journey conceptualized as one where the path being traveled can be stepped onto and away from, as study participants previously conceptualized their cancer journeys [8].

### Dementia itself CMs

This group comprised four source domain categories: a thief, a monster, an intruder, a devourer. These CMs can be grouped under the previously described conventional CM of disease as enemy [43], with the enemy in the case of dementia personified as a particularly malevolent being who invades others’ space to claim or confiscate whatever it finds there. The disease is thus conceptualized here as a creature-type entity that enters the body from the outside, rather than as a degenerative biologic process originating from within the body.

### The person with dementia’s body/brain CMs

This target domain category comprised two source domain categories: a container and a machine. These source domains accord with the conventional CMs of body as container [5] and body as machine [43], although in the case of dementia here, “body” is often specified as the brain. In the body as container CM, orientational metaphors (eg, MEs containing words such as “in,” “on,” and “across”) are frequent, conceptualizing the body as a bounded space into and out of which entities from the outside pass. In the case of the person with dementia, the container metaphor was here typically represented by a storage container from which electronic files or rows of books (ie, metaphors for memories) have been dispersed. As for the body as machine CM, the machine was typically represented as a computer or other type of circuit that is either dysfunctional or broken altogether.

### The person with dementia CMs

This target domain category comprised four source domain categories: a composite self, a person who’s losing substance, a person of low status, an animal, in which conceptualizations of fragmentation and dispersal again convey a sense of loss, this time the loss of personhood and of status and agency in the world. Noticeably absent from these findings were zombie metaphors to describe the person with dementia, which was in sharp contrast to the social construction of people with dementia as zombies previously described by Behuniak [23].

### The social experience of dementia CMs

This target domain category, the one with the smallest number of MEs, comprised three source domain categories: belonging to a different world, being in a bleak place, and putting on a pretense. The world represented by these CMs is an unfamiliar, unwelcoming one. Reminiscent of Susan Sontag’s metaphor of illness as “kingdom of the sick” [45], the belonging to a different world source domain category reflects a heightened sense of estrangement from society, to the extent that the person with dementia is conceptualized as having become a citizen of a different world. The being in a bleak place source domain category extends the image of that world to one that is a place of potential threats. Moreover, as conceptualized in the putting on a pretense source domain category, when the person with dementia does feel some sort of functioning in the “normal” world, it is because he/she is play-acting, managing somehow to project himself/herself to others as normal.

## Discussion

The rich set of CMs uncovered by this study of metaphors used by people with YOD to describe their illness experience confirms the importance of metaphor in helping people make sense of their illness [8]. Furthermore, the similarity of many of the CMs with previously reported metaphors not only of dementia but also of other illnesses supports the notion of the existence of stable sets of shared metaphors for particular domains of experience [8] (in this case illness).

In terms of metaphors related to dementia, the metaphors found in this study that align with previously reported metaphors of dementia used by people with the condition are fighting [25, 28], journeying [25, 28], loss [28], composite self [28] (labeled as “Transformation”), losing substance [25], person of low status [25, 28] (in the latter study, labeled as “Infantilization”), and animal [25]. These metaphors belong to the categories identified in the current study as conceptualizing proactive coping with dementia, interior suffering with dementia, and the person with dementia as a diminished person. Metaphors found in this study that do not align with previously reported metaphors of dementia are embodied metaphors conceptualizing the source of suffering with dementia as arising from without the person, often in the form of a natural force. Also missing from those previous studies are metaphors personifying dementia as some sort of malevolent creature. These differences in metaphors across studies might be explained by the fact that the current study aimed at a complete description of the most frequently used metaphors in the dataset, whereas the previous studies were more focused in their aims (ie, Zimmermann’s study [25] focused on how metaphors used by people with dementia both mirror and counter the metaphors used in the social discourse, and Castaño’s study [28] focused on metaphors related to basic psychologic needs of autonomy, competence, and relatedness); therefore, metaphors may have emerged but were not included in those studies because they were irrelevant to the study focus.

In terms of metaphors related to other illnesses, the metaphors found in this study are remarkably similar to those of previous studies on MND [22] and depression [18, 19]. In the study on MND, which analyzed the interview transcripts of 35 people living with MND, the authors also identified metaphors of fighting (labeled as “Battle/Fighting”), physical assault (“Self Under Attack”), and journey (“Journey Through Physical and Emotional Landscape”). Commenting on how the fight metaphors used by people with MND were rarely war-related or direct fighting-type metaphors, the authors explained this infrequency as due to MND being an incurable disease, thus making battling the disease an infeasible psychological strategy for people with MND. Interestingly, when war metaphors were used, it was by participants who had managed to survive for a longer time or who had a slower-progressing form of the disease. If we extrapolate from that interpretation, the presence of war metaphors in the current study might be explained by the fact that the progression of dementia may be slowed by factors such as life-style changes and certain treatments and, therefore, fighting while there is still the chance to delay the disease progression does remain a feasible strategy. Other metaphors in the MND study similar to those uncovered here are a sense of fracturing/fragmenting to describe suffering with MND, as well as personification of the disease as “Unseen Assailant or Thief.” In addition, contained within the “Journey Through Physical and Emotional Landscape” metaphor are metaphors related to downward movement, traveling through darkness, and being engulfed by rough sea. Thus, like the authors with YOD of the current study, the participants of the MND study used embodied metaphors related to natural forces to conceptualize their experience of the disease. Finally, a metaphor shared by both studies is that related to the social aspect of the condition, ie, the conceptualization of being excluded from society. As for the metaphor studies on depression [18, 19], both identified metaphors related to downward movement, weather, darkness, and entrapment. Charteris-Black [19] interprets these metaphors as representing the depressive person’s feelings of containment and constraint, whereas in the current study, the metaphors are interpreted as representing fear of losing control and of being overwhelmed as in the face of natural forces. Despite the differing interpretations, the close metaphoric similarity across these studies of dementia, MND, and depression is noteworthy. Future studies investigating whether the similarity extends to other neurologic/neurocognitive conditions are warranted.

### Study implications

The rich set of CMs yielded by the current study extends understanding of YOD beyond the biomedical dimension toward the psychosocial impacts experienced by those with the condition. The metaphors patients use provide health care professionals with quick access to the patient’s experience and world view [46]. Thus, enhanced awareness of the metaphors their patients use to describe their illness experience can help health care professionals develop a common language with patients to talk about the illness and to work together in its treatment [46]. It is hoped the findings of this study will be useful to health care professionals in helping their own patients to make sense of and cope with dementia. For example, health care professionals can draw on the CMs identified here to help patients forge their own metaphoric images, for example, through a dementia Metaphor Menu [47]. At the same time, health care professionals should bear in mind that metaphors do not conform to a “one size fits all”: indeed, a previous study revealed antipathy from some study participants towards the journey metaphor [16], and, as mentioned above, certain metaphors may be empowering for some people and disempowering for others.

### Study limitations

Limitations of this study include the fact that despite the efforts made to offer an analysis of metaphors representative of the majority of the authors studied, the particularity of any illness experience means the findings cannot be generalized to all persons with YOD. In addition, the data were obtained exclusively from English-language narratives. Future studies of metaphors of dementia used in texts written in other languages may yield universally shared CMs (eg, metaphors based on bodily experiences [7]) or culturally specific CMs (eg, a previous study showed that character traits are conceptualized through the taste of blood metaphor in Jordanian Arabic, but not in English [48]). A further limitation is that metaphor identification was conducted manually and by a single author, so MEs may have been missed. Despite this limitation, a rich set of CMs could be formulated from those MEs that were extracted. These CMs can be used in future research as keywords for computer-based corpus analyses to further explore the metaphors used to describe dementia and other neurologic/neurocognitive diseases.

## Conclusion

Many of the CMs of the dementia illness experience identified here are similar to those of previously reported metaphors of illness, both dementia and other conditions, thus providing support for the notion of shared metaphors across illness contexts. Moreover, in contrast to the dehumanizing and stigmatizing images hitherto used to socially construct people with dementia, the positive images inherent in the CMs of coping with dementia as being in a fight and coping with dementia as being on a journey affirm the proactivity and self-empowerment that the authors of these illness narratives maintain in the face of their condition. The CMs of dementia that have newly emerged from this study conceptualizing dementia as a force arising from without and threatening the person with dementia provide further insight into how people with dementia experience their condition. Health care professionals can draw on these metaphoric images to access their patients’ experience of dementia and to help them forge their own metaphors of the condition.

## Supporting information

S1 Table Complete final dataset of metaphoric expressions analyzed. (DOCX)
