# Supplementary material for: Metaphors of young-onset dementia in the illness narratives of those with the condition
Source: PLoS One. 2024 Dec 3;19(12):e0314717. doi: 10.1371/journal.pone.0314717 (PMC11614227; doi:10.1371/journal.pone.0314717)
Supplement: S1 Table — (DOCX) [file pone.0314717.s001.docx]

| **S1 Table: Complete Final Dataset of Metaphoric Expressions Analyzed** | | | | |
| --- | --- | --- | --- | --- |
| **Target domain** | **Source domain** | **MEs n  (%)** | **Metaphorical Expression** | **Source^a^** |
| Suffering with dementia is | being in fog/cloud | 63 (5.7) | With this disease, the sun rises and sets on a *foggy* bottom | OP76 |
|  |  |  | *a haze* at times that precludes one, among other things, from recognizing familiar faces | OP76 |
|  |  |  | I was determined to fight through *the haze* | OP81 |
|  |  |  | Like *the sea fog* that rolls in at intervals over the mud flats, the demons were disorienting | OP203 |
|  |  |  | a numbing *fog* crept in | OP203 |
|  |  |  | as I felt the numbness creep up the back of my neck like *a penetrating fog*, slowly inching to the front of my mind | OP227 |
|  |  |  | as numbness, as it does often, crept up the back of my neck and enveloped the mind. I was *fogged in* again | OP273 |
|  |  |  | the *foggy* recesses of my mind | DA225 |
|  |  |  | also, there are some days which I would liken life to being in *a fog that descends* | DA578 |
|  |  |  | the last few months had some ‘*foggy* days’ | DA578 |
|  |  |  | It became a *foggy* day | DA578 |
|  |  |  | something like *a fog* begins to descend | DA680 |
|  |  |  | the *foggy* days | DA1261 |
|  |  |  | when you are most active *the fog* descends either in patches or as an impenetrable shroud | DA1198 |
|  |  |  | what negative triggers bring down *the fog* | DA1505 |
|  |  |  | Although there are *foggy* days, they are a mixture of subtle and wretched | DA1803 |
|  |  |  | On the *foggier* days, finding the answers is clearly more difficult | DA1967 |
|  |  |  | then down comes *the fog* | DA2087 |
|  |  |  | Not only have there been more *foggy* days than experienced in 2011, 2012 or 2013 | DA2727 |
|  |  |  | *the fog* has been thicker and slower to lift | DA2727 |
|  |  |  | I do hope for as many highs and sunny days in 2015 but rather less low, thick *foggy* ones | DA2739 |
|  |  |  | enabling me to search and find positivity in *the fog* | DA2747 |
|  |  |  | I was trying to take some control over *the fog* and my emotions | DA2821 |
|  |  |  | when *the fog* descends | DA2864 |
|  |  |  | it was such a *foggy* day | DA2982 |
|  |  |  | *the fog* that descended upon me, making me very anxious | DA2982 |
|  |  |  | revealing how on the many current *foggy* days I do find it harder | DA3181 |
|  |  |  | Both yesterday and today were *foggy* days | DA3230 |
|  |  |  | *The fog* continues | DA3230 |
|  |  |  | The *foggy* days are now much patchier and sometimes more dense | DA3459 |
|  |  |  | A '*foggy* day' | DA4056 |
|  |  |  | Some have emerged to take my hand and lead me from *the fog* into the sun | DA4085 |
|  |  |  | stay on the sunlit road rather than join you by the wayside in *the fog* | DA4085 |
|  |  |  | Felt dementia got the better of me today. Thick *fog* descended | DA4168 |
|  |  |  | you, depression and Screwtape . . . lurk together as a terrible triad of *fog* and mayhem | DA4953 |
|  |  |  | I see life more clearly and*the fog* recedes | DA4998 |
|  |  |  | *the fog* of Alzheimer’s | TAT173 |
|  |  |  | my torturous, plodding demise into *a fog* of forgetfulness and death | FMW876 |
|  |  |  | Intermittent reception and *fog* | DWD106 |
|  |  |  | It feels as if there is cotton wool in my head, a sort of *fog* over my thoughts and feelings | DWD106 |
|  |  |  | I do not have enough energy to cope in *the fog* | DWD106 |
|  |  |  | the *clouded* thoughts I’ve been experiencing | UTK20 |
|  |  |  | There are days when *the fog* feels heavier than others | UTK39 |
|  |  |  | Something was cutting through *the fog* that had descended on my brain | UTK48 |
|  |  |  | My brain was *cloudy*, a fog had descended | UTK50 |
|  |  |  | My brain was cloudy, *a fog* had descended | UTK50 |
|  |  |  | *The cloud* was lifting. I looked up as if I’d been dreaming | UTK50 |
|  |  |  | Or at least on the days that *cloud over* | UTK81 |
|  |  |  | Just sit and hold on for *the mist* to clear | UTK50 |
|  |  |  | What will tomorrow bring? A clear day or a *foggy* one? | UTK81 |
|  |  |  | *A fog* descends, confusion reigns and there is no clarity from the moment I open my eyes | UTK131 |
|  |  |  | or simply waiting for *the fog* to lift | UTK131 |
|  |  |  | wait for *the fog* to lift. How did it descend so quickly? | UTK165 |
|  |  |  | wait for the fog to lift. How did it descend so quickly? Like driving on a bright day right into *a thick cloud* | UTK165 |
|  |  |  | Enough cuts through *the fog* to tell me it’s the iPad | UTK243 |
|  |  |  | see me on a *foggy* day | UTK259 |
|  |  |  | anything else will leave me *foggy* and unable to concentrate | UTK260 |
|  |  |  | those happy times cut through *the fog* | UTK266 |
|  |  |  | then *the fog* lifts and you’re back in the now | UTK290 |
|  |  |  | A good day can turn *foggy* at the turn of a page | UTK295 |
|  |  |  | *The haze* appeared at first, as if I was driving through patchy mist | UTK295 |
|  |  |  | The haze appeared at first, as if I was driving through *patchy mist* | UTK295 |
|  |  |  | When did *the fog* come down? | UTK295 |
|  | being at the mercy of water | 51 (4.6) | I will likely continue to write … until the lights dim, but other functions will continue *to ebb* | OP7 |
|  |  |  | Today’s events are a flash, fully an out-of-body encounter,*a flood* of disconnected synapses | OP13 |
|  |  |  | I felt my self-worth *ebbing like the tide* | OP57 |
|  |  |  | In Alzheimer’s, *the currents* of the disease *rise* slowly | OP67 |
|  |  |  | *The drifting* is similar to sailing in a slack wind | OP69 |
|  |  |  | You have *to row harder* with dementia, or you drift | OP73 |
|  |  |  | In Alzheimer’s, one is *in the boat* alone | OP73 |
|  |  |  | In Alzheimer’s . . . *you row* a little harder! | OP73 |
|  |  |  | The process of fighting off symptoms is exhausting, and yet exhilarating when one succeeds . . . *fighting, like salmon against a flush of water . . .* | OP85 |
|  |  |  | I was *adrift* on days, off my mooring | OP142 |
|  |  |  | I’m *listing portside* in the wake of more confusion | OP175 |
|  |  |  | I’m *. . .* *swamped* by memory loss | OP175 |
|  |  |  | I’m *adrift* in thoughts and images I can’t seem to control | OP179 |
|  |  |  | Often, I just *go with the flow* | OP179 |
|  |  |  | [Dr’s advice] has helped *to keep my head above water* | OP193 |
|  |  |  | me and others *shipwrecked i*n this fight | OP209 |
|  |  |  | Barry and I are both shipwrecked, feeling the isolation of *grounding on the shoals* of inveterate disease | OP209 |
|  |  |  | Words, images, and actions that otherwise would be caught in the prefrontal cortex *flow freely in the sewer of this disease* | OP235 |
|  |  |  | the various strategies required*to ply the currents* of this disease | OP266 |
|  |  |  | my thoughts wander through fields of memory and I *bob to the surface* suddenly and wonder for a moment who I am | LMM13 |
|  |  |  | I will *float on a tranquil sea of memory* one moment | LMM42 |
|  |  |  | be *swept away* the next by *boisterous waves* that leave me confused and uncertain | LMM42 |
|  |  |  | return me to the happy world as it was before Alzheimer's darkened my sight and *roiled* my soul | LMM143 |
|  |  |  | I decided to see the GP, as I have been concerned about my health recently – largely because of my balance. I feel as though I am *on a ship in rough seas* | DA285 |
|  |  |  | I still feel as though I’m *on a ship* | DA299 |
|  |  |  | I returned to work and pottered about feeling as if I was *on a ship in rough seas* | DA578 |
|  |  |  | I sway, wobble, and struggle to concentrate, like I’ve been on the biggest booze *cruise* imaginable | DA731 |
|  |  |  | the image of a swan being serene and fairly calm, and presenting in a certain way on the surface, but *below the surface* paddling sometimes more madly depending on which direction the current is flowing | DA1261 |
|  |  |  | I described to him*a tidal wave* of tears, fears and anxieties around dementia and life in general | DA2629 |
|  |  |  | Clinging to the flotsam and jetsam of my mind *in mid-ocean* | DA2758 |
|  |  |  | to navigate through what I’m sure will be at times*turbulent waters* | DA3724 |
|  |  |  | Although we feel my dementia has stabilised and the depression is less *tidal wave*in its impact | DA3856 |
|  |  |  | it destroys the compass and coordinates of memory, eventually leaving the mind *adrift* at sea | TAT56 |
|  |  |  | the currents and connections do their work unobstructed, unobserved, allowing *smooth passage above* | TAT59 |
|  |  |  | It wasn’t what I’d had in mind for this time in my life. But an uninvited *change of course* sometimes delivers in unexpected ways | TAT64 |
|  |  |  | I’m like the swan, *gliding above*, paddling frantically beneath | DWD102 |
|  |  |  | It seems as if I’m going *to sink* soon | DWD102 |
|  |  |  | Anxiety is *an undercurrent* in our disease | DWD111 |
|  |  |  | *the ebbs and flows* of confusion | DWD167 |
|  |  |  | all began *to engulf* me with paralyzing fears | LIL39 |
|  |  |  | I clung to that part of my duties as though they were *a life raft* | LIL81 |
|  |  |  | I was still prone to *tidal waves* of fear and apprehension | LIL108 |
|  |  |  | I feel I am *a pebble in a rapid brook* | LIL118 |
|  |  |  | my day-by-day *drifts* of attention | MLB187 |
|  |  |  | I fear the moment when *that same wave* crashes, scattering that information across the sand | UTK80 |
|  |  |  | scattering that information across the sand before *the tide sweeps* it back into a vast ocean | UTK80 |
|  |  |  | before the tide sweeps it back into *a vast ocean*, that individual wave and everything within it lost forever | UTK80 |
|  |  |  | before the tide sweeps it back into a vast ocean, *that individual wave* and everything within it lost forever | UTK80 |
|  |  |  | I shrug, happy *to go with the flow* as always | UTK268 |
|  | enduring physical assault | 47 (4.2) | *destroys* mental and physical capacity | OP2 |
|  |  |  | The disease works slowly, *destroying* the mind, stealing life in a tedious, silent dance of death | LMM5 |
|  |  |  | The disease is known*to strike* as early as thirty | LMM5 |
|  |  |  | The disease . . . appears to rest secretly inside us until its evil time arises and*a languid torture* begins | LMM5 |
|  |  |  | a man's naked struggle with the unknown on his way to trembling silence and unexplainable *torment* without the torturer | LMM7 |
|  |  |  | an insidious disease that gobbles memory and ends up *destroying* life | LMM25 |
|  |  |  | the *destructive* power of the disease over which I have no control | LMM35 |
|  |  |  | To have Alzheimer's is . . . an emotional *backbreaker* | LMM37 |
|  |  |  | my brain's eager course of *self-destruction* | LMM39 |
|  |  |  | *destructive* power of it | LMM41 |
|  |  |  | this disease's *slow, gruesome torture* | LMM45 |
|  |  |  | a brain … *being destroyed* by Alzheimer’s | LMM73 |
|  |  |  | the *destructive* behavior in the brain that is the hallmark of Alzheimer's | LMM77 |
|  |  |  | as I watch my brain *self-destruct* from Alzheimer’s | LMM83 |
|  |  |  | The style of the disease is . . . slow, writhing death, a secret *torture* in the head | LMM94 |
|  |  |  | A disease like Alzheimer's has the same power *to destroy* as a bullet or a scourge | LMM100 |
|  |  |  | Sometimes I think Alzheimer’s is like a *destructive* addiction but without any pleasure accompanying it | LMM109 |
|  |  |  | just when things were looking better Ol' Mr. Alzheimer's *hit* me | LMM120 |
|  |  |  | as my brain *murders* my sense and destroys my body | LMM127 |
|  |  |  | The disease *struck* me | LMM129 |
|  |  |  | Alzheimer's is hell-bent on *destroying* poetry and life | LMM135 |
|  |  |  | They must watch me as I lose my mind little by little,*an excruciating torture* for them as well as me | LMM135 |
|  |  |  | the disease has spared my earliest memories, allowing me to dwell in another time and place from*the tortures* of today | LMM139 |
|  |  |  | Alzheimer's is a unique and wrenching disease that *destroys* the mind | LMM142 |
|  |  |  | the diabolical disease proceeds *to kill* you slowly | LMM142 |
|  |  |  | by *destroying* what remains of your body and your life | LMM142 |
|  |  |  | It is *torture* to go though this, and slow | LMM167 |
|  |  |  | I become lost in the *destructive* power of Alzheimer's | LMM184 |
|  |  |  | this *scourge* is all right with me | LMM191 |
|  |  |  | other people whose lives are also twisted in the *torturous* claws of Alzheimer's | LMM191 |
|  |  |  | Alzheimer's can quickly *destroy* the beauty of the brain and the world it interprets | LMM199 |
|  |  |  | so that I’m better prepared, and better able to deal with what it *throws at* me | DA622 |
|  |  |  | *the hammer blow* that the diagnosis and the disease was causing me | DA1275 |
|  |  |  | to live well and remain positive despite what the disease *throws at* me | DA1433 |
|  |  |  | as you [Alzheimer’s] *drive* me into building up a presence and a constructive role | DA2077 |
|  |  |  | *damaged* though I feel | DA2747 |
|  |  |  | find ways of living as well as I can with what dementia *throws at* me | DA2792 |
|  |  |  | what dementia and life generally are ‘*throwing at* me’ | DA3265 |
|  |  |  | the trials and tribulations that dementia *throws* my way | DA4998 |
|  |  |  | So much about Alzheimer’s *throws* you into uncertainty | TAT126 |
|  |  |  | So much about Alzheimer’s  *. . . wipes out* assumptions you might have had about yourself, your mind, your capabilities, your future | TAT126 |
|  |  |  | *the ravages* of Alzheimer’s | TAT149 |
|  |  |  | a disease that is slowl*y destroying* your brain | TAT212 |
|  |  |  | what I am enduring. It’s like *water torture*: drip, drip, drip | FMW876 |
|  |  |  | I am easily reminded of my *torturous*, plodding demise into a fog of forgetfulness and death | FMW876 |
|  |  |  | It *rides roughshod over* lives, leaving tattered and shattered skeletal remains where a whole person once stood | UTK200 |
|  |  |  | the difference between the old me and the new me*hits* me so hard it leaves me without breath | UTK260 |
|  | being in a state of disorder | 27 (2.4) | *a jumble of words* awaiting order | LMM15 |
|  |  |  | the letters that form the words *squirm in their own directions* | LMM20 |
|  |  |  | Thoughts *squiggle* and writhe into sentences | LMM41 |
|  |  |  | complex ideas become *twisted* | LMM150 |
|  |  |  | mind *jumbled* in *a junkyard piled* with anxiety | LMM167 |
|  |  |  | broken thoughts from *a runaway mind* | LMM167 |
|  |  |  | Clinging to *the flotsam and jetsam of my mind* in mid-ocean | DA2758 |
|  |  |  | *thoughts tangled up* in our heads | DWD64 |
|  |  |  | we can feel exhausted, confused, *muddle*-headed | DWD99 |
|  |  |  | I am very *scrambled* in my head | DWD101 |
|  |  |  | it’s just a complete *mess* | DWD106 |
|  |  |  | inside my head is a complete *mess* | DWD106 |
|  |  |  | It is just *a muddle* of random thoughts | DWD106 |
|  |  |  | sometimes even if I hear everything you say, it sounds meaningless, just *a jumble* of sound | DWD107 |
|  |  |  | The words for those pictures seem as if they are on *a loose spinning wheel* | DWD118 |
|  |  |  | as if my shelves of neatly filed words have been swept off onto the floor, and I have to search among *untidy heaps* to find the word I am looking for | DWD118 |
|  |  |  | a world which is now experienced as increasingly *chaotic*as dementia progresses | DWD131 |
|  |  |  | this path of*disturbed* emotions and behavior | DWD132 |
|  |  |  | our thoughts and words are *tangled and confused* | DWD139 |
|  |  |  | Our inner world is *in turmoil* | DWD143 |
|  |  |  | our world around us becomes strange and our ability *scrambled* | DWD144 |
|  |  |  | this strange*mixed-up* world being created by our brain damage | DWD148 |
|  |  |  | the layer of emotions. … This becomes increasingly *scrambled* | DWD162 |
|  |  |  | as my train of thought traveled too fast for the tongue, and *on too many tracks simultaneously* | LIL117 |
|  |  |  | I was noticeably *scattered* in looking after myself and my possessions | MLB111 |
|  |  |  | there’s a feeling I can liken to a fine necklace being all *tangled up* | UTK131 |
|  |  |  | everything becomes *scrambled* | UTK205 |
|  | being in a void | 16 (1.4) | In *deep space*, as in Alzheimer’s | OP191 |
|  |  |  | *the void* of Alzheimer’s | OP305 |
|  |  |  | I’m *suspended* in time, *hanging* by a rotting thread of memory | LMM59 |
|  |  |  | takes my mind’s eye to the trail’s edge of this Alzheimer’s path and I can’t help but look down. It’s an unsettling glimpse into *the abyss* | TAT158 |
|  |  |  | We feel as if we are hanging onto a high cliff, above a lurking *black hole* | DWD98 |
|  |  |  | A *black hole* opened up behind me, and in front of me | DWD105 |
|  |  |  | I am like *a high-wire act* in a circus, needing lots of concentration otherwise I’ll fall off | DWD119 |
|  |  |  | that *yawning chasm* of fear that opened up at that moment of diagnosis | DWD158 |
|  |  |  | I . . . sit alone in my home . . .  I am *suspended* | LIL112 |
|  |  |  | see my plateau as encouragement, a teeny ray of light in *a darkening void* | LIL135 |
|  |  |  | It was a  . . . *black hole* | UTK48 |
|  |  |  | going *over the edge*into someone I don’t recognise | UTK81 |
|  |  |  | before we topple *off the end* altogether | UTK121 |
|  |  |  | What will happen to me when I go *over the edge* into that person I don’t know? | UTK123 |
|  |  |  | see myself move closer and closer towards *the edge of the cliff* | UTK285 |
|  |  |  | when I’m close enough to look over and see *a blank below* | UTK285 |
|  | being in darkness | 16 (1.4) | The most disturbing symptoms in *my private darkness* are the visual misperceptions | OP30 |
|  |  |  | as if a light in my brain had been shut off. I was overcome by *the darkness* of not knowing where I was or who I was | OP227 |
|  |  |  | The stress of Alzheimer’s, for those diagnosed, and for their caregivers, is deadening deep into *the darkness* | OP228 |
|  |  |  | the unfathomable *darkness* of this disease | OP272 |
|  |  |  | That’s Alzheimer’s. A *light goes off*, and one goes into a rage because it’s *dark* | OP318 |
|  |  |  | the happy world as it was before Alzheimer's *darkened my sight* and roiled my soul | LMM143 |
|  |  |  | there is *light at the end of the tunnel* | DA1650 |
|  |  |  | My life now is a bit like climbing mountains and looking at the view from the summit, alongside scrambling along pot holes into deep caverns which are *dark* and need *a light shining* to guide me | DA2060 |
|  |  |  | my awareness of other things going on around me *dimmed* | TAT72 |
|  |  |  | there are ‘*windows of clarity*’ which we must take advantage of | DWD99 |
|  |  |  | other days, the sun does shine, and *by this light I see life more clearly* | DA4998 |
|  |  |  | the *curtains had fallen across* behind me | DWD107 |
|  |  |  | The *black curtain falls down* behind me as I desperately try to search for some recollection | DWD108 |
|  |  |  | I was *blindfolded*, no longer able to function in society | DWD156 |
|  |  |  | Frantic, I tried to recollect the client’s name and what we were discussing. I could not. I had not the slightest *glimmer* | LiL39 |
|  |  |  | see my plateau as encouragement, a teeny *ray of light* in a *darkening* void | LIL135 |
|  | being trapped | 15 (1.4) | saw myself outside the box of dementia, yet felt *trapped* within it | OP48 |
|  |  |  | a man *entangled* in his own death | LMM30 |
|  |  |  | a man *entangled* helplessly in his own unwanted death | LMM55 |
|  |  |  | Alzheimer's has . . . captured my dreams. Now I am certain there is *no place to hide* | LMM162 |
|  |  |  | I have Alzheimer’s. But I’m *stuck* with it | TAT8 |
|  |  |  | If I can speak, I am not sick. This is the big dilemma, the ‘*Catch 22*’ of dementia | DWD48 |
|  |  |  | an attitude that isolates us, into *a separate, walled compartment of dementia* | DWD142 |
|  |  |  | we are not far away or lost, but *trapped* by an inability to communicate and to think clearly | DWD148 |
|  |  |  | I was mentally *mired in quicksand* | LIL8 |
|  |  |  | It was like being in *a maze* | LIL39 |
|  |  |  | Living in *the labyrinth* can be lonely and frightening | LIL120 |
|  |  |  | Surely that much love can’t just disappear? It must stay *trapped* inside instead | UTK143 |
|  |  |  | the love must just be *locked away* behind those sad, glazed eyes | UTK145 |
|  |  |  | I was lost inside. Screaming to *get out*. It was terrifying | UTK245 |
|  |  |  | Is that the real me *trapped* inside | UTK284 |
|  | being pulled down | 15 (1.4) | the right side of my brain is intact. The left side … is, at times, *in a free fall* | OP7 |
|  |  |  | the powerful pull of the Alzheimer’s *downward spiraling trajectory*never let up | OP312 |
|  |  |  | tied to a teetering mind that had begun a slow *descent* into silence | LMM3 |
|  |  |  | The disease is known to strike as early as thirty, but only a tiny minority*falls*in its clutches before the mid-sixties | LMM5 |
|  |  |  | Eventually there is*a descent* into silence | LMM5 |
|  |  |  | my *descent* into the arms of Alzheimer’s | LMM78 |
|  |  |  | I feel I have *fallen down* a deep well of anxiety | LMM107 |
|  |  |  | Sometimes a disturbing dream, a mood, a moment of forgetfulness or *a depressive dip*draws me in | TAT158 |
|  |  |  | as I *slip* farther *into* the fog of Alzheimer’s | TAT173 |
|  |  |  | I suddenly lost my ability to read and*spiraled down* until doctors were able to turn the situation around | TAT184 |
|  |  |  | She sees me as basically a good person who has*fallen* ill | FMW644 |
|  |  |  | in the end you*slip into* a coma and die | DWD177 |
|  |  |  | And so my *downward slide* continued | MLB4 |
|  |  |  | my *descent* into the post-cerebral realm for which I am headed | MLB4 |
|  |  |  | I am sad and*deflated* | UTK94 |
|  | trying not to lose hold | 14 (1.3) | I was trying desperately *to hold on to* who I was for as long as possible | OP124 |
|  |  |  | I mus*t cling to* optimism and avoid depression | LMM26 |
|  |  |  | *Clinging to* the flotsam and jetsam of my mind in mid-ocean | DA2758 |
|  |  |  | what I do have are WORDS, HOPE and SPIRIT, and those I will *hold on to* for as long as I possibly can | DA4105 |
|  |  |  | I wanted *to hold onto* my belief in overcoming the medical model | DWD19 |
|  |  |  | for Marie’s personable letters. They helped me *hang on* when I felt there was *nothing to hang onto* | LIL75 |
|  |  |  | I managed *to cling to* a small shred of independence | LIL81 |
|  |  |  | I *clung to* that part of my duties | LIL81 |
|  |  |  | I also *clung to* that belief and was nurtured by it | LIL93 |
|  |  |  | I must *hang in* and hang tough | LIL113 |
|  |  |  | One of our founding group members died. The rest of us remain, *clinging to* our plateaus | LIL139 |
|  |  |  | *holding tight on to* every nerve | UTK31 |
|  |  |  | *losing a grip* on what makes me me | UTK81 |
|  |  |  | That is the thought that *clings on* through the storm | UTK278 |
|  | being under someone else’s control | 11 (1.0) | thoughts and images I can’t seem to control. They *rule me* | OP179 |
|  |  |  | before long it will have *complete and absolute control over* you | LMM108 |
|  |  |  | a mental or physical reminder of *its absolute power over* me | LMM108 |
|  |  |  | the new normal that Alzheimer’s would *impose* | TAT184 |
|  |  |  | Was I some kind of *ventriloquist’s dummy*? | DWD65 |
|  |  |  | By forming support groups, we patients can *take the reins* of our life *up in our own hands again* | LIL137 |
|  |  |  | before the dementia *takes the decision out of my hands* | UTK89 |
|  |  |  | That’s *not me in control* – it’s dementia | UTK259 |
|  |  |  | It’s just *seduced me into* working with it, not against it | UTK259 |
|  |  |  | with this disease any suffering will go on as long as my brain *dictates* | UTK285 |
|  |  |  | Powerless to live the way I want to, and so I *scrape back control* whenever I can | UTK285 |
|  | being fast-forwarded, slowed down, or paused | 8 (0.7) | early-onset Alzheimer’s; it’s a death *in slow motion*. A freeze frame at times | OP7 |
|  |  |  | early-onset Alzheimer’s . . . *A freeze frame* at times | OP7 |
|  |  |  | This is an unfinished story of a man dyin*g in slow motion* | LMM6 |
|  |  |  | The style of the disease is *slow motion* | LMM94 |
|  |  |  | I was witnessing my own neurodegeneration *in slow motion* | TAT76 |
|  |  |  | as if I’d *fast-forwarded* into a patch preview of a later-stage Alzheimer’s | TAT114 |
|  |  |  | it’s very much like I’m fading away and dying *in slow motion* | FMW427 |
|  |  |  | Everything *slowed* right down—time and actions | UTK295 |
|  | things slipping away/disappearing | 61 (5.5) | I started asking questions, trying to fill in *the blanks* | OP80 |
|  |  |  | The mind was *blank* | OP80 |
|  |  |  | Alzheimer’s  . . . an alternate definition of which is “a place where people and things *disappear without a trace*” | OP193 |
|  |  |  | Yesterdays *disappear*, except those long ago | LMM5 |
|  |  |  | meaning and substance *disappear* quickly | LMM31 |
|  |  |  | events not yet *blotted* from my mind | LMM32 |
|  |  |  | Thoughts squiggle and writhe into sentences that *disappear*before | LMM41 |
|  |  |  | I have only a few seconds to capture a thought before it *disappears* from my mind | LMM48 |
|  |  |  | Ideas *evaporate* like snowflakes on a warm roof | LMM48 |
|  |  |  | I sense reality *slipping away*, and words become *slippery sand* | LMM89 |
|  |  |  | Language *is gone* | LMM142 |
|  |  |  | Memory *disappears* | LMM142 |
|  |  |  | the only things of that time to remain are memories, and now they *slip away* | LMM143 |
|  |  |  | I may be unable to catch the idea or thought before it *disappears* | LMM150 |
|  |  |  | I am not dying, just *disappearing* before your eyes | LMM157 |
|  |  |  | shards of memory kept *disappearing* suddenly | LMM159 |
|  |  |  | the words that sometimes *slip through* the crevices in my mind | DA4125 |
|  |  |  | the occasional *blanks* recalling names and such | TAT63 |
|  |  |  | *cognitive gap* | TAT70 |
|  |  |  | I’d have difficulty *filling in* missing words from context | TAT163 |
|  |  |  | this memory may *fade* into obscurity as my dementia progresses | FMW330 |
|  |  |  | as my memory *fades*with each breath I take | FMW330 |
|  |  |  | it’s very much like I’m *fading away* | FMW427 |
|  |  |  | more recent memories and thoughts *slip away* from me like a helium balloon | FMW669 |
|  |  |  | My life . . . looks like it’s going to *fade away* at the hands of the devastating disease of Alzheimer’s | FMW784 |
|  |  |  | We were . . . then *fading into* tiredness and blankness in-between | DWD52 |
|  |  |  | My mind was often *blank* | DWD98 |
|  |  |  | we have no recollection. It is just *a blank* for the past | DWD98 |
|  |  |  | as our brain *disappears* | DWD100 |
|  |  |  | a stream of ideas  . . . there one moment, *totally gone* the next | DWD101 |
|  |  |  | Yesterday or today, last week or the week before, are *a blank* | DWD106 |
|  |  |  | my mind goes *blank* | DWD106 |
|  |  |  | the thought does not come back later — it is *gone for good* | DWD118 |
|  |  |  | when an idea comes into our head, because if we wait, it will *disappear* | DWD139 |
|  |  |  | Cognition is *fading* | DWD161 |
|  |  |  | the disease eats away at the brain, so that slowly it *disappears* | DWD177 |
|  |  |  | get dressed without many*memory blanks* | MLB108 |
|  |  |  | I searched my memory and found it *blank* | LIL8 |
|  |  |  | my *waning* abilities | LIL99 |
|  |  |  | indicative of *slipping* capacities | LIL100 |
|  |  |  | my memory *slips* | LIL108 |
|  |  |  | As *my grip* upon the present *slips* | LIL109 |
|  |  |  | the determination to endure rather than simply give up and *fade away* | LIL137 |
|  |  |  | This was totally different. This was totally *blank* | UTK1 |
|  |  |  | a *vanished* verb | UTK1 |
|  |  |  | an *absent* adjective | UTK1 |
|  |  |  | desperate not to let a single thought *slip through* the net | UTK20 |
|  |  |  | the word I need next is lost, and instead there is *a blank* in my mind where it should be | UTK28 |
|  |  |  | Those days are becoming more and more frequent, the focus *fading* | UTK41 |
|  |  |  | people I’ve worked with for years walk into the room and *a blank* meets my mind, *a space inside* where their name usually fitted neatly | UTK41 |
|  |  |  | And then she asks for those three words she’d told me at the beginning of the session, and again, they’ve *slipped away* without me noticing | UTK44 |
|  |  |  | It was *a complete blank* | UTK48 |
|  |  |  | I can’t follow the recipe . . .  I turn the page and *poof, it’s gone* | UTK62 |
|  |  |  | I will store it to memory. I won’t let it *slip away* | UTK69 |
|  |  |  | there is that sense of urgency, to fit in a future before it *disappears* forever | UTK81 |
|  |  |  | if a thought pops into our heads we’re allowed to share it straight away rather than risk it *vanishing* | UTK119 |
|  |  |  | when there’s *a space* where their name should be | UTK143 |
|  |  |  | it’s not items that *disappear*, but our most precious memories | UTK145 |
|  |  |  | There’s *a blank* where other Christmases once existed | UTK230 |
|  |  |  | *The blank* that has become an all-too-familiar visitor to my mind | UTK241 |
|  |  |  | a numbness, *an emptiness* replaces my busy, creative mind | UTK295 |
|  | loss | 53 (4.8) | *losing* a sense of self | OP57 |
|  |  |  | *loss* of self | OP206 |
|  |  |  | *loss* of filter | OP235 |
|  |  |  | Meaning is *lost* in a hurried moment | LMM15 |
|  |  |  | a word *lost* in confusion | LMM15 |
|  |  |  | *loss* of language | LMM35 |
|  |  |  | At any moment I may *lose* my train of thought | LMM68 |
|  |  |  | *losing* another neurotransmitter and the story that went with it | LMM68 |
|  |  |  | I am *losing* what has been stored | LMM119 |
|  |  |  | I regress, *losing* adult characteristics | LMM119 |
|  |  |  | you *lose* your sense of being human | LMM142 |
|  |  |  | you slowly *lose* yourself | LMM142 |
|  |  |  | I am *losing* precious memory | LMM150 |
|  |  |  | I am *losing* familiarity with myself | LMM172 |
|  |  |  | eventual*loss* of nearly everything human beings value | LMM181 |
|  |  |  | the emotional impact of*the loss*or struggle as the disease advances | TAT9 |
|  |  |  | easy for me *to lose* the thread that I want to write about | TAT225 |
|  |  |  | am I *losing* myself? | FMW330 |
|  |  |  | *losing* the super-fast, super-smart me | DWD17 |
|  |  |  | we *lose* the inter-connectedness of our thread of life | DWD98 |
|  |  |  | I have *lost* that vibrancy, the buzz of interconnectedness, the excitement and focus I once had | DWD102 |
|  |  |  | I have *lost* immediacy | DWD106 |
|  |  |  | as if I have *lost* the filter in my brain | DWD114 |
|  |  |  | *loss* of self | DWD130 |
|  |  |  | progression of *losses*that occur in dementia | DWD130 |
|  |  |  | each successive *loss* | DWD130 |
|  |  |  | *loss*of self-esteem | DWD131 |
|  |  |  | *lose* the thread of what we say | DWD139 |
|  |  |  | anticipatory grief at *loss* of self | DWD143 |
|  |  |  | *losing* ourselves as well as others | DWD143 |
|  |  |  | We are *losing* our cognitive self | DWD168 |
|  |  |  | you *lose* all sorts of abilities | DWD177 |
|  |  |  | I was afraid I “had *lost* my mind” | LIL9 |
|  |  |  | *losing* my intelligence | LIL53 |
|  |  |  | before I *lost* all the instructions | LIL69 |
|  |  |  | each additional *loss* of memory, and concentration | LIL97 |
|  |  |  | *losing* my train of thought altogether | LIL99 |
|  |  |  | the word I need next is *lost* | UTK28 |
|  |  |  | why is something *missing*? | UTK30 |
|  |  |  | ten minutes *lost* | UTK39 |
|  |  |  | I *lost* time again today | UTK39 |
|  |  |  | something more cherished than any of my possessions would be *lost* | UTK71 |
|  |  |  | I *lost* yesterday | UTK130 |
|  |  |  | but when I begin to speak, the words are *lost* | UTK138 |
|  |  |  | I feel again the sense of *loss* within myself | UTK138 |
|  |  |  | I’m *losing* a little bit of me every day | UTK155 |
|  |  |  | the square I know so well but which had been *lost* in an instant | UTK166 |
|  |  |  | I’m *losing* the past | UTK182 |
|  |  |  | not just the joy of cooking that’s now *lost*, but eating too | UTK220 |
|  |  |  | The retirement that I’d once planned has been *lost* to a disease that I hadn’t asked for | UTK238 |
|  |  |  | If I don’t use it, I will *lose* it | UTK245 |
|  |  |  | scared of *losing* the last bit of me | UTK255 |
|  |  |  | Words are often *lost* now | UTK255 |
|  | fragmentation | 32 (2.9) | as short-term memory began *to disintegrate* | OP161 |
|  |  |  | an ever-slowing *breakdown* of mind and body | OP163 |
|  |  |  | a *breakdown* of synapse | OP163 |
|  |  |  | Alzheimer’s *breaks* the mind, then the body *down* | OP206 |
|  |  |  | These memories become the last *remnants*of my search for who I am | LMM42 |
|  |  |  | My life is turning into a dun-colored *kaleidoscope* | LMM89 |
|  |  |  | My mind is starting *to break down* | LMM137 |
|  |  |  | I thought I was*breaking into pieces* | LMM159 |
|  |  |  | *shards of memory*kept disappearing suddenly | LMM159 |
|  |  |  | mind jumbled in a junkyard piled with anxiety and *broken thoughts* from a runaway mind | LMM167 |
|  |  |  | The other image I use is of*a picture which a hole punch has made holes in,* sometimes few, sometimes many | DA120 |
|  |  |  | My long-term memory was *largely intact*, although *some holes* were starting to appear | TAT73 |
|  |  |  | I am cognitively *intact* enough to comprehend the material he is writing and to detect subtle jokes | FMW330 |
|  |  |  | Life has become *a fragmented kaleidoscope* of problems | DWD98 |
|  |  |  | It’s like I have *a patchwork mind* | DWD120 |
|  |  |  | *My brain is like a sieve*, with facts falling through *the holes*. I need to read fast . . . to stop them all falling through *the cracks* | DWD120 |
|  |  |  | our crisis of identity and *our fragmentation* | DWD130 |
|  |  |  | as we cope with *shattered thoughts* and fragmented selves | DWD138 |
|  |  |  | as we cope with shattered thoughts and *fragmented selves* | DWD138 |
|  |  |  | Our sense of self is *shattered* | DWD156 |
|  |  |  | At the centre of our being lies the true self … this is what remains *intact*, it is what makes us who we really are | DWD162 |
|  |  |  | The precious string of pearls, of memories, that is our life, *is breaking*, the pearls are being lost | DWD169 |
|  |  |  | My world was *disintegrating* | LIL67 |
|  |  |  | I managed to cling to a small *shred* of independence | LIL81 |
|  |  |  | I feared losing my last *shred* of dignity | LIL82 |
|  |  |  | my *fraying memory* caused me stress | MLB3 |
|  |  |  | my *unraveling mind* | MLB76 |
|  |  |  | my mind is already *buckling under* the vagaries of matter | MLB187 |
|  |  |  | as my brain *disintegrates* | MLB245 |
|  |  |  | That’s why I feel *broken* | UTK62 |
|  |  |  | the part of my brain that can type fluently is *still intact* | UTK181 |
|  |  |  | my thoughts became more *fragmented*, like wisps, not fully formed | UTK295 |
|  | unsuccessful searching/finding | 13 (1.2) | I *find* the word I was *searching for* | OP1 |
|  |  |  | Words . . . before they were *retrievable* and now they are not | LMM31 |
|  |  |  | the frustrating *search* to communicate | LMM184 |
|  |  |  | I tried desperately *to find* the word for the place where we had been meeting | DWD64 |
|  |  |  | I can’t *find* the memories when I want to | DWD105 |
|  |  |  | meeting you happens too quickly for me *to search* through my disjointed memory and *find* a label for you | DWD109 |
|  |  |  | It is as if my shelves of neatly filed words have been swept off onto the floor, and I have *to search* among untidy heaps *to find* the word I am *looking for* | DWD118 |
|  |  |  | I *searched* my memory and *found* it blank | LIL8 |
|  |  |  | the word I’d been so desperately *searching for* | UTK28 |
|  |  |  | missing the conversation because I’m *sifting through* it for the mention of a name | UTK41 |
|  |  |  | finding it impossible *to retrieve* the right words to join in or keep up | UTK44 |
|  |  |  | Dementia . . .  might have made it harder for me to grasp hold of the ones I’m *searching for*in time to finish a sentence | UTK181 |
|  |  |  | The words are often too hard *to find* | UTK283 |
| Coping with dementia is | being in a fight *(War)* | 212 (19.1) | They are soldiers on the front lines in *this lonely fight* | OP6 |
|  |  |  | I try to stay locked in, *as a missile is on a target* | OP7 |
|  |  |  | *The battle* is numbing | OP30 |
|  |  |  | You must *know your enemy*—*study with military precision—to fight your enemy* | OP50 |
|  |  |  | I was in full-throttle denial, responding to *a five-alarm call to arms*: protect my wife, my children, myself, my business, and my friends | OP53 |
|  |  |  | *a surrender to* numbness | OP69 |
|  |  |  | My life has become *a strategy* | OP75 |
|  |  |  | I have*a formidable enemy*—my mind | OP75 |
|  |  |  | In Alzheimer’s, brain cells in charge of short-term memory are *losing the war* | OP89 |
|  |  |  | I returned to the party, feeling like *a time warrior* | OP176 |
|  |  |  | me and others shipwrecked in*this fight* | OP209 |
|  |  |  | *the steady onslaught* of Alzheimer’s | OP231 |
|  |  |  | when the light in the brain goes out, … often without notice, and synapse is *firing wildly like a sniper* | OP256 |
|  |  |  | we often *withdraw to the bunker* | OP256 |
|  |  |  | retreating to our inner selves, *keeping our heads down below the tracer bullets* | OP256 |
|  |  |  | “Al” was bearing down, and I was *demoted to the rank of private* | OP266 |
|  |  |  | a gende*r under siege* | OP303 |
|  |  |  | I am at the beginning of the disease's *onslaught* | LMM117 |
|  |  |  | It seems*a weak armada* to defend against eager memory destroyers working in my brain | LMM179 |
|  |  |  | It seems a weak armada to defend against eager *memory destroyers* working in my brain | LMM179 |
|  |  |  | So it is that Alzheimer's begins *its conquest* | LMM268 |
|  |  |  | *the slow march* of this disease | LMM401 |
|  |  |  | Although *subtle in attack,* Alzheimer's | LMM655 |
|  |  |  | *the onslaught* of the disease | LMM1803 |
|  |  |  | *the march* of Alzheimer’s | LMM2032 |
|  |  |  | *my battle* with Alzheimer’s | LMM2464 |
|  |  |  | *the war* in my brain | LMM2621 |
|  |  |  | I am *on the losing side in a battle* with Alzheimer's | LMM2621 |
|  |  |  | my brain was *under siege* from Alzheimer's | LMM2669 |
|  |  |  | Alzheimer's *at a quick march* | LMM2669 |
|  |  |  | It is *a battle* with little purpose | LMM2796 |
|  |  |  | this is *one battle I will not win* without a miracle | LMM2796 |
|  |  |  | As the brain is slowly devoured and gradually *succumbs* | LMM3149 |
|  |  |  | I sense you see this as*a conflict* | DA1198 |
|  |  |  | if so, *some battles I have won* | DA1198 |
|  |  |  | you encourage apathy, and then use this as *a weapon* to bring about decline | DA1198 |
|  |  |  | You have few *allies* and mine are growing in number | DA1208 |
|  |  |  | I do feel somewhat *‘bulletproof’* at the moment | DA1433 |
|  |  |  | *my armoury* against you | DA1646 |
|  |  |  | I have a growing network of *allies* who stand alongside me | DA1647 |
|  |  |  | even our country’s leader is *lined up in opposition to* you | DA1651 |
|  |  |  | In addition to *my human allies* I now have another in the form of medication: galantamine | DA1652 |
|  |  |  | the daffodil not only heralds spring, but *resistance to* your cold clutches | DA1654 |
|  |  |  | For too long there have been no new drugs *to defeat* your evil approaches | DA1654 |
|  |  |  | your evil *approaches* | DA1654 |
|  |  |  | I try *to outmanoeuvre and outwit it* | DA1995 |
|  |  |  | How can I see you as *the enemy* when you are part of  me | DA2078 |
|  |  |  | I do still feel *bulletproof* | DA2080 |
|  |  |  | *encased and protected by a shield of armour*from your insidious *advances* | DA2080 |
|  |  |  | there is hope and ways in which we can *keep you at bay* | DA2093 |
|  |  |  | I have many *conflicts* now inside my head | DA 2323 |
|  |  |  | especially if I put it *above the parapet* to ‘be shot at’ | DA2323 |
|  |  |  | which results in feeling exposed, lonely and *a target* | DA2324 |
|  |  |  | I used to think I was *bulletproof and armour plated* | DA2632 |
|  |  |  | What has alarmed me is that you have *recruited y*our toxic cousin, depression, *into your armoury* | DA2753 |
|  |  |  | *To combat* this, I sense the need for those around me, that is, friends and family, to be as positive as possible | DA2753 |
|  |  |  | with their support and love, I see you *retreat* | DA2756 |
|  |  |  | despite your attempts at *sabotage* I do my best to ensure that this hope becomes a reality | DA4079 |
|  |  |  | I have no *suit of armour* against you, no immunity, no tricks, but what I do have are WORDS, HOPE and SPIRIT | DA4120 |
|  |  |  | *gains against* you since you came to reside inside my head | DA4967 |
|  |  |  | especially among those physicians *on the front lines* with patients who might benefit | TAT7 |
|  |  |  | no reliable way to slow down*the inexorable march* of this disease | TAT23 |
|  |  |  | amp up my brain’s *defenses against* it | TAT87 |
|  |  |  | as the brain itself is *under siege* | TAT90 |
|  |  |  | a mark of *my battle with* Alzheimer’s | TAT120 |
|  |  |  | I felt utterly helpless being unable to slow down*the inexorable march* of this disease | TAT125 |
|  |  |  | These fairly simple choices become *an organized counterattack against* Alzheimer’s | TAT126 |
|  |  |  | Music turns nostalgia into a kind of *neurological resistance to* a disease that takes no prisoners | TAT149 |
|  |  |  | a disease that *takes no prisoners* | TAT149 |
|  |  |  | One thing we can do is to take *the private battle*public | TAT181 |
|  |  |  | However personal*my medical battle against* Alzheimer’s | TAT182 |
|  |  |  | I *am battling* its power to avert our gaze | TAT182 |
|  |  |  | *battling to* survive the decline of dementia | DWD222 |
|  |  |  | *battling* a terminal illness | DWD479 |
|  |  |  | *a constant battle to overcome* the fear of these later stages | DWD540 |
|  |  |  | This continuing *battle to overcome* such negative attitudes | DWD680 |
|  |  |  | we are *battling* the decline | DWD694 |
|  |  |  | *my battle with* Alzheimer’s | DWD718 |
|  |  |  | *battling with* our losses and decline | DWD1111 |
|  |  |  | This *assault* to our functioning | DWD1890 |
|  |  |  | The second is *the battle*we have with what I call the ‘disease of society’ | DWD2238 |
|  |  |  | this *latest battle*for existence | DWD2457 |
|  |  |  | *My battle with* little memory losses and balance | LIL142 |
|  |  |  | *fight my battles* with me | LIL723 |
|  |  |  | the phone, with its faceless voices, has become*an enemy* | LIL142 |
|  |  |  | I don’t want to just sit here and wait for this disease *to make its march on* my mind | UTK103 |
|  |  |  | as the disease *makes its march on* our brain | UTK200 |
|  |  |  | To allow this disease*to make its march*even sooner? | UTK238 |
|  |  |  | for every clever way I find *to combat* dementia | UTK249 |
|  |  |  | many days it feels that I am *fighting a losing battle*– and I am | UTK285 |
|  | *(Race/Chase)* |  | a walking nightmare in which you can’t escape the bogeyman no matter how fast you *run* | OP28 |
|  |  |  | Alzheimer’s is *a marathon* against time | OP28 |
|  |  |  | keep running *to outpace* this disease | OP28 |
|  |  |  | this disease that ultimately will *overtake* me | OP28 |
|  |  |  | "Get out of my car!" Brendan demanded, yet another *passing of the baton* in my progression | OP167 |
|  |  |  | I had *to sprint*, a full-out panic dash, to avoid capture at sundown | OP203 |
|  |  |  | I had to sprint, a full-out panic *dash*, to avoid capture at sundown | OP203 |
|  |  |  | The demons were advancing … Faster and faster they *chased* | OP204 |
|  |  |  | The demons were advancing … I *beat them* with every ounce of will in me | OP204 |
|  |  |  | My life, once a distance run, is now *a race* for survival | OP204 |
|  |  |  | So I *run from* the demons of illusions, confusion, rage, and ongoing depression | OP204 |
|  |  |  | My daily running routine has become symbolic of *the chase*, *a race* ultimately I will lose | OP204 |
|  |  |  | Two guys trying *to beat the curve* | OP210 |
|  |  |  | Alzheimer’s *pursues* at a calculating pace | OP300 |
|  |  |  | I am writing in a panic,*racing against* an insidious disease | LMM25 |
|  |  |  | I am *running after* thoughts all day | LMM48 |
|  |  |  | The crippling effects of Alzheimer's were *closing in on* me | LMM128 |
|  |  |  | I will do everything I can *to keep one step ahead* of you | DA4085 |
|  |  |  | the inevitable decline that *catches up with* even the most diligently monitored patient | MLB8 |
|  |  |  | the realization that I am probably coming toward my *final stretch* | LIL87 |
|  |  |  | not if I want to win one day after the next, not if I want *to stay one step ahead of this disease* | UTK258 |
|  | *(General)* |  | we will need every ounce of hope in *fighting* Alzheimer’ s | OP2 |
|  |  |  | You get *knocked down*, you get back up. Again and again. You find a way to win | OP6 |
|  |  |  | I’m *getting even with* Alzheimer’s | OP8 |
|  |  |  | The *fight* against this disease consumes me | OP29 |
|  |  |  | Most diseases attack the body, bu*t Alzheimer’s attacks* the mind, then the body | OP31 |
|  |  |  | [My doctor] has urged me … to stop *assaulting* Alzheimer’s *head-on*. “You can’t win in *a head butt*… You just have to learn to dance with it!” | OP35 |
|  |  |  | “I can deal with this. … I can *fight* it” | OP49 |
|  |  |  | So, at twilight, I’m back *on the mat* with the monster | OP76 |
|  |  |  | *fighting off s*ymptoms is exhausting . . | OP85 |
|  |  |  | It is a forceful *fight* for clarity, one that I win more than I lose now | OP85 |
|  |  |  | It is a forceful fight for clarity, one that I *win more than I lose now* | OP85 |
|  |  |  | vile diseases that *attack* the soul as well as the body | OP211 |
|  |  |  | Many … choose to deflect the relentless in-your-face *assault* of these demons | OP229 |
|  |  |  | these demons, hoping*to stare* them *down* for as long as possible | OP229 |
|  |  |  | It’s a lonely, numbing gaze, *a confrontation* that sometimes one cannot win | OP229 |
|  |  |  | the unremitting *struggle*to stay in the moment | OP275 |
|  |  |  | in *a fight* against a demon of a disease | OP315 |
|  |  |  | I’m *in the ring* with the tiger | TAT8 |
|  |  |  | the emotional impact of the loss or *struggle* as the disease advances | TAT9 |
|  |  |  | *holding my own against* Alzheimer’s | TAT10 |
|  |  |  | the growing pandemic of Alzheimer’s disease and*attack* it with the same determination . . . we are seeing now in response to Covid-19 | TAT126 |
|  |  |  | I was basically deciding to take *the “fight”* public | TAT182 |
|  |  |  | My hope is that the world will soon wake up to the explicit threat of the growing pandemic of Alzheimer’s disease and *attack* it with the same determination, ingenuity and cooperation we are seeing now in response to Covid-19. | TAT216 |
|  |  |  | what I’m *contending with* | DA662 |
|  |  |  | I respect your tenacity in not giving up in your efforts to*get the better of* me | DA731 |
|  |  |  | these are the earliest of days in our relationship and I hope *to win more exchanges than I lose* with you over the coming years | DA741 |
|  |  |  | in some you have *gained an upper hand* | DA1198 |
|  |  |  | although your *victories* will leave a bitter taste, they will be short-lived | DA1198 |
|  |  |  | I will *contest* this with you through remaining busy, active, engaged | DA1198 |
|  |  |  | I have others helping me to surround myself with a *protective* shell | DA1208 |
|  |  |  | my toolkit *to outwit* you | DA1640 |
|  |  |  | the effort *to get the better of* you | DA1650 |
|  |  |  | I will do EVERYTHING I can to help *to defeat* you | DA1650 |
|  |  |  | I cannot *fight* you, in the long term I cannot *beat* you, but I will not cower or stay silent in your presence | DA1650 |
|  |  |  | Nor will I refrain from my best efforts *to outwit* you | DA2077 |
|  |  |  | One area where you and I *wrestle*is around the use of language | DA2077 |
|  |  |  | but I am *fighting* you | DA2087 |
|  |  |  | this *fight* is certainly worth doing | DA2087 |
|  |  |  | I have been*struggling*lately | DA2715 |
|  |  |  | I feel constantly that I have *to defend* myself | DA2727 |
|  |  |  | you’ve *won some rounds* | DA2747 |
|  |  |  | but I still feel like I’m still *winning the bout* | DA2747 |
|  |  |  | it was only through Liz and the five students that we were able *to defeat* you | DA2747 |
|  |  |  | the Alzheimer’s Society, a charity whose mission is *to defeat* you | DA2758 |
|  |  |  | feel a sense of release from what dementia *confronts* me with | DA2864 |
|  |  |  | six key words as heading to help *defeat* you | DA3427 |
|  |  |  | A book which you know will *challenge* you, dementia, to the core of your existence | DA4075 |
|  |  |  | One piece which inspires me and helps me*confront* you | DA4085 |
|  |  |  | dementia *got the better of* me today | DA4168 |
|  |  |  | *struggled*to cope with dealing with shops | DA4674 |
|  |  |  | of a man's *naked struggle* with the unknown | LMM7 |
|  |  |  | but to me it is hardly a start at*licking* the malady | LMM86 |
|  |  |  | This is *a big win* for positive mental attitude | LMM86 |
|  |  |  | I will have to wipe the frown off my face and *show my teeth* more often | LMM86 |
|  |  |  | *beaten* by the inevitable progress of a mind-stealing illness | LMM151 |
|  |  |  | The disease *has wrested* me *from* the command of my skills and even the secrets of sleep | LMM162 |
|  |  |  | the *struggle* to reclaim lost words | LMM184 |
|  |  |  | this daily *struggle* | DWD13 |
|  |  |  | in our *struggle* with dementia | DWD13 |
|  |  |  | this *struggle* to survive | DWD19 |
|  |  |  | you are really*fighting* this disease at the moment | DWD19 |
|  |  |  | as someone *struggling* with this terminal illness | DWD47 |
|  |  |  | my *struggle* with my illness | DWD48 |
|  |  |  | we experience*a defeat* of spirit and of hope | DWD53 |
|  |  |  | *overcoming* the trials of dementia | DWD79 |
|  |  |  | We face a daily *struggle*to cope | DWD98 |
|  |  |  | it’s like *struggling* to live in a fog | DWD99 |
|  |  |  | Everything is confusing, and *the struggle*is exhausting to the point of extreme tiredness | DWD99 |
|  |  |  | our *struggles*each day to cope | DWD100 |
|  |  |  | It is such*a struggle* to get my thoughts and ideas out | DWD101 |
|  |  |  | *the struggle*is getting to the point where I feel too exhausted to keep going like this . . . *a struggle*every day. …so hard*to struggle*to cope each day | DWD102 |
|  |  |  | life is full of*struggles* and complexities | DWD103 |
|  |  |  | the person with dementia *struggles*to interpret | DWD131 |
|  |  |  | each day is such *a struggle* | DWD136 |
|  |  |  | two burdens from our disease. The first is the *struggle with* the illness itself | DWD142 |
|  |  |  | in the midst of *a struggle* to retain a sense of who we are | DWD157 |
|  |  |  | Daily life is*a struggle* | DWD163 |
|  |  |  | Our passage towards this choice will be*a struggle*of feeling to achieve healing | DWD167 |
|  |  |  | *the struggle with* dementia | DWD169 |
|  |  |  | I might be*defying* this dementia | DWD179 |
|  |  |  | daily *struggles* with this disease | DWD180 |
|  |  |  | find joy in small *victories* | LIL62 |
|  |  |  | I experienced a soul-searing*confrontation* with my deficits | LIL101 |
|  |  |  | I was going *to outwit* any impending diagnosis | UTK38 |
|  |  |  | staring hard at the photograph, determined *to outwit* my fading brain | UTK69 |
|  |  |  | dementia is nothing more than a trick of my mind, and I can *outwit*it | UTK72 |
|  |  |  | Dementia isn't *winning* in that room—we are | UTK119 |
|  |  |  | must be a way of *outwitting* this disease | UTK126 |
|  |  |  | all the journeys I take with a smile on my face, knowing I have *outwitted* Alzheimer’s again | UTK127 |
|  |  |  | I don’t want to acknowledge the days when Alzheimer’s *wins* | UTK130 |
|  |  |  | *outwitting* the panic that always threatens to descend with dementia | UTK146 |
|  |  |  | Just leaving out the negative word ‘worse’ would give hope that I could find some way *to outwit* the part of my brain that is no longer working as it should | UTK170 |
|  |  |  | my independent nature – which has so far *defeated* this disease | UTK175 |
|  |  |  | no harm, on some occasions, *letting* the disease *win* once in a while | UTK194 |
|  |  |  | I’m smiling as I return downstairs and flick the kettle on, *dementia outsmarted*once more | UTK210 |
|  |  |  | I*beat* dementia by still being organised | UTK248 |
|  |  |  | *to outwit* a disease | UTK256 |
|  |  |  | a disease I know ultimately will *win* | UTK256 |
|  |  |  | if I want *to win*one day after the next | UTK258 |
|  |  |  | it *claims minor victories* every day | UTK258 |
|  |  |  | my head was*struggling*to keep up with the words in front of me | UTK295 |
|  | being on a journey | 114 (10.3) | my own *journey* with Alzheimer’s | OP6 |
|  |  |  | *The journey* through Alzheimer’s is a marathon, if one chooses to run it | OP28 |
|  |  |  | The drifting is similar to sailing in a slack wind. In Alzheimer’s, one doesn’t move fast, but *the journey* is soporific | OP69 |
|  |  |  | if I was heading to Pluto … for *the final trip* | OP173 |
|  |  |  | caught again in the mirror of infinity—seeing the past, confused in the present, and preparing *to head for* Pluto, Sedna, and beyond for the final staging | OP187 |
|  |  |  | Like the Pluto spacecraft itself, we’re all *on a journey* to unknown places | OP202 |
|  |  |  | My life has now become a series of anecdotes, revelations in *this journey* | OP202 |
|  |  |  | we’re*on the same train* | OP321 |
|  |  |  | *Along the way*, I’ve also discovered | TAT2 |
|  |  |  | nature itself is an impartial guide*on this journey* | TAT2 |
|  |  |  | Sometimes the thought of the years ahead with Alzheimer’s feels especially daunting, a towering monolith, a sheer rock face. At those times, I think of Beacon Rock, the gift of *switchbacks* and the power of *one step at a time* | TAT19 |
|  |  |  | For the most part, *sailing* is *not primarily about reaching a destination*. It’s all about the joy of *getting there* | TAT66 |
|  |  |  | Often the joy isn’t even about *getting where you planned*, but more about being where you are | TAT69 |
|  |  |  | takes my mind’s eye to*the trail’s edge of this Alzheimer’s path*and I can’t help but look down | TAT158 |
|  |  |  | And in conversations with those who are*on their own path* with this disease | TAT176 |
|  |  |  | easy to forget that I am *headed for* disaster in the future | TAT191 |
|  |  |  | helps motivate me *to stay the course* each day | TAT191 |
|  |  |  | *this journey* has thus far taken me on many ups and downs | DA240 |
|  |  |  | My life now is a bit *like climbing mountains* and looking at the view from the summit, alongside *scrambling along pot holes into deep caverns* which are dark and need a light shining to guide me along *the slippery route* | DA2060 |
|  |  |  | life with you [Alzheimer’s]  is *not an easy ride* | DA2087 |
|  |  |  | a bit *like driving along a road* when you *travel* in and out of clear road and patches of fog | DA3459 |
|  |  |  | to surmount the obstacles and hurdles that are going to be *in my way* | DA3724 |
|  |  |  | *the dementia pathway* | DA4020 |
|  |  |  | and stay *on the sunlit road,* rather than join you *by the wayside* in the fog | DA4085 |
|  |  |  | *The journey* with dementia has to date taken seven and a half years | DA4943 |
|  |  |  | when I look back it seems*a long, long way from there to here* | DA4943 |
|  |  |  | I wonder how much longer I am *to carry on travelling* | DA4943 |
|  |  |  | It has been*a long way from there to here* | DA4963 |
|  |  |  | hopefully, ‘dear Alzheimer’s’, there is still *a long way to go* | DA4963 |
|  |  |  | *to travel this journey* with dementia | DWD9 |
|  |  |  | I am still here, and it has been quite *a journey* of understanding, of seeing more clearly who I am now, who I am becoming, and who I will be when I die | DWD10 |
|  |  |  | *a journey* of living positively with dementia | DWD10 |
|  |  |  | discovering*a journey* into the centre of self | DWD10 |
|  |  |  | Each person with dementia is *travelling a journey* deep into the core of their spirit | DWD11 |
|  |  |  | I was well into *my journey* with the disease | DWD17 |
|  |  |  | when my exciting *‘roller-coaster’ journey* really started | DWD18 |
|  |  |  | his sincerity and willingness to begin *this journey* alongside us | DWD34 |
|  |  |  | *this journey* with dementia | DWD35 |
|  |  |  | *the long journey* . . . between diagnosis and the end stage | DWD39 |
|  |  |  | *the journey* of living with dementia each day | DWD39 |
|  |  |  | what it felt like to be*on the journey* from diagnosis to death | DWD39 |
|  |  |  | dementia is not just an end stage, it is *a journey*, from diagnosis to death | DWD40 |
|  |  |  | there are many steps *along the way* | DWD40 |
|  |  |  | after diagnosis, there is usually *a journey* of several years, in which we are battling the decline | DWD48 |
|  |  |  | still being able to speak after years of living *the journey* of dementia | DWD49 |
|  |  |  | the people living *this journey* from diagnosis through to death with dementia | DWD60 |
|  |  |  | you cannot empathise, you cannot provide the care we need *to travel this traumatic road* | DWD60 |
|  |  |  | And we were each just a bit further into *our individual journeys* with dementia | DWD72 |
|  |  |  | adapting my care as I *journey into* areas of different need | DWD74 |
|  |  |  | living this life *in the slow lane* | DWD88 |
|  |  |  | making *a long journey* of diagnosis and treatment, and of self discovery and reflection | DWD88 |
|  |  |  | *the emotional and spiritual journey that I was travelling* | DWD90 |
|  |  |  | the beginning of *a long slow journey* of change | DWD97 |
|  |  |  | as *we journey* further into our decline | DWD101 |
|  |  |  | *our journey* of decline | DWD101 |
|  |  |  | as *we journey* deeper into the centre of our being, into our spirit | DWD123 |
|  |  |  | the whole *gamut* of emotions we will experience *along the journey* of their [sic] disease | DWD130 |
|  |  |  | feeling of horror at what might*lie ahead* | DWD131 |
|  |  |  | *this path* of disturbed emotions and behavior | DWD132 |
|  |  |  | to hope for a new life *in the slow lane* | DWD134 |
|  |  |  | We can learn new things, in*this journey* to focus on what is important | DWD136 |
|  |  |  | a new life *in the slow lane* | DWD143 |
|  |  |  | the person *travelling the journey* with dementia | DWD152 |
|  |  |  | I*travel this journey* of dementia | DWD155 |
|  |  |  | There is *no stage in this journey*at which you must abandon all hope of connecting with me, as we can remain linked through our spirits — not our minds | DWD155 |
|  |  |  | It’s *a journey* into ceasing to be | DWD157 |
|  |  |  | It has been*a long journey*for me since 1995 to learn how to live positively each day with my diagnosis of dementia | DWD158 |
|  |  |  | My *journey w*ith dementia has been *a journey* of self-discovery about who I really am | DWD158 |
|  |  |  | Dementia is often thought of as death by *small steps*, but we must ask ourselves what is really dying | DWD158 |
|  |  |  | Hasn't the person with dementia*reached* that place of 'now', of existing actively in the present? | DWD158 |
|  |  |  | I believe that people with dementia are making*an important journey* from cognition, through emotion, into spirit | DWD159 |
|  |  |  | what really remains throughout *this journey* is what is really important, and what disappears is not important | DWD159 |
|  |  |  | As *we journey* towards our spiritual self, as our outer masks decrease, our inner self increases. Cognition is fading, emotion and spirit are increasing | DWD161 |
|  |  |  | We can be strengthened in our spirit as we *make this journey* with dementia | DWD161 |
|  |  |  | in *this journey* towards my true self, with dementia stripping away the layers of cognition and emotion, I’m becoming who I really am | DWD161 |
|  |  |  | It is *a journey* with simplicity, one that moves from the outer mask of cognition | DWD162 |
|  |  |  | *the journey* of dementia | DWD162 |
|  |  |  | My *journey* into the spirit is freeing | DWD163 |
|  |  |  | I am surviving *this journey* with dementia and, rather than fighting the disability, I am adapting to it in a dance | DWD163 |
|  |  |  | By accepting*this journey* of change and adaptation | DWD166 |
|  |  |  | we can dance with dementia and choose a new life *in the slow lane* | DWD166 |
|  |  |  | as we celebrate this new life *in the slow lane* | DWD166 |
|  |  |  | We are *on a path to* healing, through feeling and acknowledging our fear, anxiety, and the ebbs and flows of confusion | DWD167 |
|  |  |  | we can work towards creating a new future, of being a survivor. *Our passage* towards this choice | DWD167 |
|  |  |  | creating a new future of life *in the slow lane* | DWD168 |
|  |  |  | It is rather my eternal life that is important, and that remains in my spirit through and beyond *this journey* with dementia | DWD168 |
|  |  |  | walking with you on *that journey* from diagnosis to death | DWD170 |
|  |  |  | *This journey* of survival, of uncovering the inner spirit | DWD170 |
|  |  |  | is *a journey* of letting go, and finding inner peace | DWD170 |
|  |  |  | my *journey* has taken me into an ever deeper and more trusting relationship with God | DWD170 |
|  |  |  | *this journey* from diagnosis to death | DWD172 |
|  |  |  | I had created a detailed agenda to keep me *on track* | MLB3 |
|  |  |  | my descent into the post-cerebral realm for which *I am headed* | MLB4 |
|  |  |  | Its answer to Murdoch’s question “When are we going?” is “*We have already left*. It is only a matter of time befor*e we arrive*” | MLB66 |
|  |  |  | Since I myself am *walking the teetering plank toward* the neither-dead-nor-alive identity of someone with dementia | MLB231 |
|  |  |  | [the Alzheimer patient] they *travel through the dangerous twists and turns* of the labyrinth | LIL(1) |
|  |  |  | I felt I was *on a trip to* never-never land | LIL19 |
|  |  |  | *The bridge I was crossing* was precarious enough due to its missing planks | LIL82 |
|  |  |  | Without someone *to walk this labyrinth*by my side | LIL114 |
|  |  |  | without the touch of*a fellow traveler*who truly understands my need of self-worth | LIL114 |
|  |  |  | how can I endure the rest of*this uncharted journey*? | LIL114 |
|  |  |  | someone walking *the same bridge we are walking* | LIL118 |
|  |  |  | Bridge walking is hazardous when there are planks missing and *the traveler must step with caution* | LIL118 |
|  |  |  | *fellow traveler* on our bridge of missing planks | LIL122 |
|  |  |  | I realize*how far I have come* psychologically, if not intellectually | LIL125 |
|  |  |  | The tests indicate I am still basically *in a holding pattern*, on a “plateau,” that favorite of Alzheimer buzzwords | LIL135 |
|  |  |  | much too concerned, and desperately so, with *what lies* between where we are today and the end | LIL139 |
|  |  |  | not the end which occupies our nightmares and daytime panics . . . It is how we *reach* that end | LIL139 |
|  |  |  | We devote our time, attention, and energies to *the road we are on, not the destination* | LIL139 |
|  |  |  | the racing mind and thoughts of *the road ahead* | UTK105 |
|  |  |  | ‘Dementia has to start somewhere,’ I explain. ‘It doesn’t always mean the end stages, and I’m just an example of someone *at the start of the journey* | UTK284 |
|  |  |  | Can I *go on*? | UTK284 |
|  |  |  | I want to turn back to the now, and not have *to go down that road* | UTK284 |
|  |  |  | Do I want to continue long enough to see myself *move closer and closer towards* the edge of the cliff? | UTK285 |
| Dementia itself is | a thief | 44 (4.0) | Alzheimer’s, which slowly *robs* one of self | OP2 |
|  |  |  | knowing the disease ultimately would *rob* that from me | OP124 |
|  |  |  | that’s what this disease does. It *robs* | OP293 |
|  |  |  | quickly *one is robbed* of the most precious memories | OP311 |
|  |  |  | The disease works slowly, destroying the mind, *stealing* life | LMM5 |
|  |  |  | a disease that slowly *squeezes* the life *from* you | LMM8 |
|  |  |  | Alzheimer's *deprives* us both of life | LMM44 |
|  |  |  | a *mind-stealing* illness | LMM151 |
|  |  |  | As disease subtly *steals* these sweet nuggets of the past | LMM194 |
|  |  |  | what you are seeking *to take from* me | DA1208 |
|  |  |  | you *wrest* the words from my tongue | DA2758 |
|  |  |  | for fear of you *whisking away* what I wish to say | DA2758 |
|  |  |  | I am cognizant of what Alzheimer’s *has taken away* | TAT145 |
|  |  |  | Alzheimer’s *robs* us of the future we hoped for | TAT180 |
|  |  |  | our silence allows Alzheimer’s *to rob* us of even more | TAT180 |
|  |  |  | It is *stealing* not only my memories and my ability to function | FMW669 |
|  |  |  | It is *stealing* the legacy I want to leave for my wife and daughters | FMW669 |
|  |  |  | this mystery illness that *robs* us of who we think we are | DWD11 |
|  |  |  | Alzheimer’s is meant to *take away* your ability to learn new things | DWD25 |
|  |  |  | each of us had progressive illnesses that would *take* our ability *away* bit by bit | DWD71 |
|  |  |  | with dementia *stripping away* the layers of cognition and emotion | DWD161 |
|  |  |  | the way it *takes away* the mind bit by bit | DWD178 |
|  |  |  | That’s what dementia *steals*, the future you imagined all laid out in front of you | UTK47 |
|  |  |  | develop a disease that would *steal* memories from me | UTK71 |
|  |  |  | it’s a *thief* in the night, *stealing* precious pictures from our lives while we sleep | UTK71 |
|  |  |  | it is inevitable that dementia will *steal* these memories from me | UTK72 |
|  |  |  | for what *it’s stolen*, for what *it’s about to steal* | UTK79 |
|  |  |  | Dementia *hasn’t yet stolen* all the knowledge | UTK87 |
|  |  |  | before the dementia *takes* the decision *out of my hands* | UTK89 |
|  |  |  | what dementia has *stripped away from* me | UTK114 |
|  |  |  | its monstrous mission, *stealing* memory upon memory | UTK130 |
|  |  |  | no amount of love we feel for someone can protect against *the theft* of our recognition of them | UTK143 |
|  |  |  | The film shows how memory is *stripped away* so indiscriminately | UTK143 |
|  |  |  | what Alzheimer’s can give, not just *take away* | UTK150 |
|  |  |  | this disease can *steal* the past, the present and the future | UTK166 |
|  |  |  | Dementia may have*stolen* the words from my mouth | UTK181 |
|  |  |  | I resent dementia for *stealing* the image of my future that I’d so carefully created in my head | UTK186 |
|  |  |  | as if the disease has just reached in there and *plucked* it *out* | UTK186 |
|  |  |  | That my disease, and everything so terrible that it *steals* from me | UTK193 |
|  |  |  | It *steals* from us memories | UTK200 |
|  |  |  | hate my disease, not for what it’s *stealing* from me now | UTK200 |
|  |  |  | I hate my disease, not for what it’s stealing from me now, but what it plans *to take from* my girls | UTK200 |
|  |  |  | Another thing dementia is *stripping from* me is my emotions | UTK250 |
|  |  |  | this is a memory that dementia will never *steal* from me | UTK294 |
|  | a monster | 33 (3.0) | this *monster* disease | OP1 |
|  |  |  | *this demon* prowling like Abaddon | OP8 |
|  |  |  | *These demons,* I keep telling myself, don’t know who they’re fucking with! | OP22 |
|  |  |  | a walking nightmare in which you can’t escape *the bogeyman*no matter how fast you run | OP28 |
|  |  |  | I had unleashed *a monster* | OP127 |
|  |  |  | *This monster* will be slayed only when we collectively understand its extensive reach | OP160 |
|  |  |  | *the demon* Alzheimer’s | OP173 |
|  |  |  | as angels visit and*the demons* chase | OP202 |
|  |  |  | *The demons* were chasing | OP203 |
|  |  |  | *the demons* were disorienting | OP203 |
|  |  |  | *The demons* were advancing | OP204 |
|  |  |  | So I run from*the demons* | OP204 |
|  |  |  | I’m moving backwards into Alzheimer’s, into the hands of a pack of forbidding *demons* | OP204 |
|  |  |  | *The monsters* have followed me here | OP206 |
|  |  |  | *The demons* were back again months later with a vengeance | OP207 |
|  |  |  | the relentless in-your-face assault of *these demons* | OP229 |
|  |  |  | How is anyone going to know when *the demon* is coming? | OP300 |
|  |  |  | saw*the demon*coming years ago | OP300 |
|  |  |  | Alzheimer’s pursues at a calculating pace … *this prowling demon* that seems to be locked on to a gender, affecting women far more than men | OP300 |
|  |  |  | This disease is *a cruel monster* | OP312 |
|  |  |  | in a fight against*a demon* of a disease | OP315 |
|  |  |  | surrounded with memories, events not yet blotted from my mind by *the eager beast* in my brain gobbling time in both directions | LMM32 |
|  |  |  | This *evil* disease sleeps on the edge of my consciousness, always there to remind me of its *wicked* strength over me | LMM46 |
|  |  |  | this *hellish* disease | LMM69 |
|  |  |  | the way Alzheimer's wiggles *its evil fingers* at you | LMM108 |
|  |  |  | The person in me who lives on until natural death occurs is only a shadow left by *the deadly laugh of Alzheimer's* | LMM117 |
|  |  |  | I am staring *a monster* in the face | LMM126 |
|  |  |  | as the *diabolical* disease proceeds to kill you slowly | LMM142 |
|  |  |  | *the dark beast* that dwells in my brain | DA58 |
|  |  |  | [you, depression and Screwtape] *you and your dark allies* | DA4953 |
|  |  |  | You tell me you are*a servant of death*, and at some point your master will take me, but it is not in your power to decide when that will be | DA4963 |
|  |  |  | it drags behind me like *a specter* | MLB127 |
|  |  |  | claiming more brain cells for its *monstrous* mission | UTK130 |
|  | an intruder | 21 (1.9) | unless something is done to subdue *the insidious intruder* | OP74 |
|  |  |  | Alzheimer's begins *its conquest* | LMM12 |
|  |  |  | an *insidious* disease that gobbles memory | LMM15 |
|  |  |  | the peculiar way Alzheimer's sneaks in and begins *taking over* | LMM25 |
|  |  |  | as*the disease claims more mental territory* | LMM45 |
|  |  |  | as Alzheimer’s *works its way through* my brain | LMM76 |
|  |  |  | the illness's *intrusion* | LMM94 |
|  |  |  | the mental *contamination* of Alzheimer's | LMM160 |
|  |  |  | as I watch Alzheimer’s *take over* | LMM162 |
|  |  |  | as Alzheimer's *gains control of* my mind and body | LMM194 |
|  |  |  | it is so close to you, *gaining control* of your brain | DA1640 |
|  |  |  | my own early-stage Alzheimer’s as it *stakes out* its slowly growing presence in my brain | TAT1 |
|  |  |  | the inevitable *encroachments* of Alzheimer’s | TAT1 |
|  |  |  | depending on who you are and how Alzheimer’s *lands* in your life | TAT138 |
|  |  |  | I recognise at times that you try *to infiltrate* this network | TAT181 |
|  |  |  | allowing the unexpected*to invade* my life | DWD19 |
|  |  |  | this disease that has *taken over* my brain | UTK112 |
|  |  |  | *claiming* more brain cells | UTK130 |
|  |  |  | my head feels . . . as if it doesn’t belong to me – and it doesn’t, it’s *given over to* the disease | UTK130 |
|  |  |  | It’s not me: it’s this cruel disease *invading* my head | UTK134 |
|  |  |  | this disease follows me everywhere, *bleeds into* every moment | UTK282 |
|  | a devourer | 16 (1.4) | It is analogous to the prototypical arcade game Pac-Man . . . this Pac-Man in slow motion *consumes* brain cells, one by one | OP24 |
|  |  |  | I am being *gobbled up* in time | LMM20 |
|  |  |  | an insidious disease that *gobbles* memory | LMM25 |
|  |  |  | the eager beast in my brain*gobbling* time in both directions | LMM32 |
|  |  |  | Alzheimer’s is the closest thing to *being eaten alive* slowly | LMM41 |
|  |  |  | before the bumbling and forgetfulness *consumes me* | LMM42 |
|  |  |  | Alzheimer's silently hollows the brain and fills it with death. Even memory *is burned* | LMM60 |
|  |  |  | Even memory is burned, leaving *nothing to suck* for internal substance | LMM60 |
|  |  |  | Alzheimer’s*burns* the familiar and turns the world into an uncertain, frightening place | LMM78 |
|  |  |  | the disease *eats away at* the brain | DWD177 |
|  |  |  | It *eats its way into* your brain, into the frontal and temporal lobes | MLB66 |
|  |  |  | It *feasts on* Wernicke’s area | MLB66 |
|  |  |  | *gorges itself on* Broca’s area | MLB66 |
|  |  |  | before too long dementia will likely have *eroded* that recently implanted memory | MLB184 |
|  |  |  | my biggest fear is that dementia will be *eating* a large enough portion of my already non-normal-appearing and -functioning neurons | MLB234 |
|  |  |  | On those days I can feel the disease in my head, like it’s *eating away at* all that is good in there | UTK130 |
| The person with dementia’s body/brain is | a container | 54 (4.9) | I thought of my brain *as a large depository*, a dumping ground of sorts, *a large storage bin* for stashing a cornucopia of politics, current events, sports, trivia, and points of view | OP23 |
|  |  |  | My brain was once *a file cabinet*, carefully arranged in categories, but at night as I sleep, it’s *as if someone has ransacked the file*s, dumping everything onto a cluttered floor. Before I get out of bed each morning, I have to pick up the “files” and arrange them in the correct order | OP32 |
|  |  |  | *My Elmer’s bottle is empty* | OP187 |
|  |  |  | the sinuous, peripatetic Scarecrow in . . . The Wizard of Oz, for those of us with Alzheimer’s—the disease is a ride over the rainbow. *Our heads are full of stuffin’* | OP266 |
|  |  |  | The disease . . . appears to*rest secretly inside us* | LMM5 |
|  |  |  | to have this disease sprout *in my body* | LMM44 |
|  |  |  | It is hard to live *in a grown-up body* and have the mind of a child | LMM98 |
|  |  |  | As the brain is slowly devoured and gradually succumbs, *turning the body into an empty vessel* | LMM207 |
|  |  |  | remembering and writing are more than difficult; they are *cold receptacles emptied of content* | LMM207 |
|  |  |  | an increasingly flawed *filing system of my brain* | DA225 |
|  |  |  | things have not been *going into the old grey matter* as readily as they did previously | DA313 |
|  |  |  | I said, ‘It’s a bit like those ball bearings and that physics motion where one ball bearing knocks out another ball bearing, or two ball bearings knock two ball bearings out. Is this called a Newton’s Cradle?’ As I tried to cling onto what information I was being told, the new piece of information was knocking the old piece *out of my brain* | DA680 |
|  |  |  | I have many conflicts now *inside my head* | DA2322 |
|  |  |  | a lovely day in the sunshine*—both outside and inside my head* | DA2982 |
|  |  |  | the tau-containing neurofibrillary tangles spreading on the PET scan of my brain. Those horses are stomping their feet, anxious *to get out of the barn* | TAT185 |
|  |  |  | listening to *the music within* | DWD13 |
|  |  |  | these views are no longer *in my head* | DWD57 |
|  |  |  | thoughts tangled up *in our heads* | DWD64 |
|  |  |  | thoughts tangled up in our heads, crossed wires misfiring, and odd words *coming out* | DWD64 |
|  |  |  | My body was exhausted and my mind stretched beyond its meagre *capacity* | DWD88 |
|  |  |  | such a struggle *to get* my thoughts and ideas *out* | DWD101 |
|  |  |  | *inside my head* is a complete mess | DWD106 |
|  |  |  | a thought *comes in* | DWD106 |
|  |  |  | That thought won’t *pop into my head* again | DWD106 |
|  |  |  | I pointed to the outline of squares on the paper shoji screen behind us. I said “Each one of *these boxes is like one of my memories, kept locked up behind a little door.* My keys are lost, and I have this two dimensional feeling, of a blank wall of memory doors behind me for which I have lost the keys. Give us clues so we can join in with your memory and do not be upset if the key no longer fits the lock, or the store of memories behind the door has faded. | DWD108 |
|  |  |  | The noise or motion feels like an egg beater *in my head* | DWD114 |
|  |  |  | my head, scrambling what is *in there* and putting a static sound or visual screen over what is coming in | DWD114 |
|  |  |  | my head . . . putting a static sound or visual screen over what is *coming in* | DWD114 |
|  |  |  | I feel somehow ‘scoured out’ *inside my head* … | DWD115 |
|  |  |  | *In our head* a string of pictures has formed | DWD118 |
|  |  |  | *as if my shelves of neatly filed words* have been swept off onto the floor, and I have to search among untidy heaps to find the word I’m looking for | DWD118 |
|  |  |  | the wrong word *comes out* | DWD118 |
|  |  |  | when an idea comes *into our head* | DWD139 |
|  |  |  | Shouting simply distresses us – for me it feels as if you are hitting my head, causing even more confusion *inside there* | DWD140 |
|  |  |  | happening *inside our heads* | DWD148 |
|  |  |  | my *emptied-out-head*-attached-to-my-life-clinging-body | MLB236 |
|  |  |  | I had them *back in my memory*, but had to consciously recall them | LIL33 |
|  |  |  | A line from the screenplay “Inherit the Wind” *came into* my thoughts | LIL80 |
|  |  |  | spark a memory still alive and retrievable *within my brain* | LIL98 |
|  |  |  | The sensation of *a head half-filled with* cotton wool has continued for months | UTK9 |
|  |  |  | Why won’t the next process *come into my head*? | UTK30 |
|  |  |  | I feel empty *inside* | UTK63 |
|  |  |  | all that information I have stored inside *my mental filing cabinets* | UTK80 |
|  |  |  | each time the thought of one *creeps in* | UTK81 |
|  |  |  | You might like to think of the memory of someone with dementia as a bit *like a bookcase* as tall as I am. A cheap, mass-produced, flat-pack bookcase. This bookcase is full of books that contain factual memories. The top shelf – the ones you have to stand on your tiptoes to reach – hold very recent memories, such as what you had for breakfast this morning. By your shoulders are books from perhaps your fifties, the ones that all of us are used to reaching out and taking from the shelf any time we like – no effort, hardly any strain. And by your knees are books from your twenties. And then you get all the way down to your feet, where just beyond the tips of your toes you’ll find books from your childhood. Having dementia rocks your bookcase from side to side, and it’s always the books at the top that fall first, jumbling everything else up, so sometimes what you think of as your most recent memories will come from further down the bookcase, earlier in your life | UTK97 |
|  |  |  | There is another part of the brain, another bookcase, separate from the first, more flimsy one. This bookcase is sturdy; it is *your emotional bookcase*. When dementia attempts to rock this one side to side, as if the two versions of you – before and after – are two tectonic plates that collide beneath the solid ground, *this bookcase is stronger, more resilient, so the contents will be safer for longer*. Even though you may forget that your friends or family visited recently – because that book comes off your factual bookcase – what stays with you are the feelings you had of love, happiness and comfort when they were near. You may forget what you did, what you spoke about or that they even popped by, but you know you feel safe and happy when you see them | UTK98 |
|  |  |  | We agree that if a thought pops *into our heads* | UTK119 |
|  |  |  | On those days I can feel the disease *in my head*, like it’s eating away at all that is good *in there* | UTK130 |
|  |  |  | It doesn’t just hit my ears – it smashes into them, rolling around *inside my brain* | UTK184 |
|  |  |  | snippets of conversations now feeling like a swarm of bees *inside my mind* | UTK185 |
|  |  |  | I’ve noticed this time my brain can’t hold multiple plans *inside my brain* | UTK205 |
|  |  |  | The words . . . take too long *to come out* | UTK283 |
|  |  |  | while words lose themselves on their way *out of my mouth* | UTK259 |
|  |  |  | a flashback to another era, a file taken down from *the shelf* from so long ago | UTK290 |
|  | a machine | 44 (4.0) | Daily exercise and writing are my succor, helping me *reboot* and reduce confusion | OP7 |
|  |  |  | a collective long-term memory, *the hard drive* of one’s life | OP10 |
|  |  |  | a combined therapy that serves *to reboot* the brain | OP23 |
|  |  |  | one can *re-circuit the brain* to receive and transmit information | OP25 |
|  |  |  | I think of my brain today, once a prized possession, *as* *an iPhone*: still *a sophisticated device*, but one that *freezes up*, *shuts down* without notice, *drops calls*, *pocket dials* with random or inappropriate conversation, and has *a small battery that takes forever to charge* | OP29 |
|  |  |  | As the brain shrinks, it instinctively makes decisions … on what functions to power and what functions to power down *to preserve fuel* | OP31 |
|  |  |  | My brain . . . may be conserving power . . . a sort of *cerebral brownout*, akin to a calculated reduction in big city voltage to prevent electrical blackout | OP32 |
|  |  |  | My brain, a.k.a. *HAL* | OP32 |
|  |  |  | the interruptions of a brain gone awry, *a flickering light whose plug is loose in the socket* | OP69 |
|  |  |  | with the brain *in the “on” position* | OP72 |
|  |  |  | Consider the 1982 movie *Tron* in which a computer programmer is transported inside the software world of *a computer mainframe* and engages terrifying sequencers in an effort to get back. That’s my world today | OP74 |
|  |  |  | I must focus on the 5 W’s: the who, what, where, when, why, and how of life, *as if rebooting my faithful MacBook Pro* before tossing the covers and organizing *the scattered files* of my mind | OP74 |
|  |  |  | Some say the brain acts as *computer*; others suggest it’s a symphony orchestra. The brain is probably a little of both | OP75 |
|  |  |  | a daily exercise of body and mind to engage the brain, as if *pulling the chord to a cold chainsaw*. You gotta rip at it | OP75 |
|  |  |  | Like the Griswold house, *the lights* in my head blink; they are full on, off, back on, then off again, on again | OP84 |
|  |  |  | after *restarting* my brain at the gym | OP137 |
|  |  |  | the brain functions *like a laptop frozen in a software pinch*, displaying a disturbing rainbow-colored icon that spins at high speeds, declaring the computer is not engaged. Everything is shut down | OP187 |
|  |  |  | My brain, on this day, is not responding. The *rainbow icon* is spinning again | OP187 |
|  |  |  | I run to*jumpstart my brain*at the end of the day, a process *akin to crank starting a chainsaw* after it has sat overnight on a New England deck in February | OP204 |
|  |  |  | Running helps *to reboot* the mind | OP204 |
|  |  |  | Running for me *flicks the light back on* | OP204 |
|  |  |  | My progression of Alzheimer’s advances as *the lights go faint in the brain* | OP204 |
|  |  |  | neurons had just *misfired* | OP234 |
|  |  |  | *Lights in my brain* that *had been flickering* in this early stage were now *at full power* | OP270 |
|  |  |  | I used lists and calendar notes more often – my *private backup system* | TAT70 |
|  |  |  | two incidents of absent-mindedness in a single day disturbed me. Both were*glitches* in ordinary kitchen routines I’d done a million times | TAT71 |
|  |  |  | impossible for me to override this isolated *glitch* in my neural processing | TAT74 |
|  |  |  | slow the progress of the disease and*amp up* my brain’s defenses against it | TAT87 |
|  |  |  | Pressing alternate neural pathways into service or forging new ones, this reserve acts something *like a backup generator* to keep *the lights on* when *the main power source is failing* | TAT89 |
|  |  |  | If a song I haven’t heard for fifty years or more comes on the radio, it immediately*tags* a memory from the time I used to listen to it | TAT148 |
|  |  |  | now I’m *hotwiring* my brain for names | TAT188 |
|  |  |  | Alzheimer’s *corrupts the connections* and threatens to disable the system | TAT221 |
|  |  |  | Alzheimer’s corrupts the connections and threatens *to disable the system* | TAT221 |
|  |  |  | thoughts tangled up in our heads, *crossed wires misfiring*, and odd words coming out | DWD64 |
|  |  |  | My speed of *information processing* is far too slow | DWD108 |
|  |  |  | When my brain becomes overloaded and fatigued, it’s *like a short circuit*, and my brain cuts out | DWD115 |
|  |  |  | this neuro-*glitch*-from-hell | LIL123 |
|  |  |  | each night my brain is *deleting files* as I sleep | UTK102 |
|  |  |  | If only we could pick and choose *the files that get deleted* | UTK102 |
|  |  |  | there is little in my brain to help me through; it’s *as if it’s been emptied overnight in my dreams, rebooted and restored to factory settings* | UTK131 |
|  |  |  | A *short circuit* in my brain | UTK166 |
|  |  |  | keeping those *circuits firing in my brain* | UTK247 |
|  |  |  | the sudoku I do every morning *to kick-start* my brain | UTK247 |
|  |  |  | my brain *plugging* into the bits it can remember | UTK255 |
| The person with dementia is | a composite self | 38 (3.4) | One never knows who’s going to show up in the early stages of this disease: *the new me*, or *the old me*? Will I be on or off today? | OP228 |
|  |  |  | retreating to *our inner selves* | OP256 |
|  |  |  | I wonder who I am; how am I defined by myself, and by others – who is *the real ME* in deMEntia? | DA493 |
|  |  |  | I just seemed to be more of ‘*my old self’*again | DWD 18 |
|  |  |  | very hard for us to be *who we once were*, so let us be *who we are now* | DWD126 |
|  |  |  | *the* vibrant, energetic *person you once knew is now someone* who doesn’t participate as actively as they once did | DWD133 |
|  |  |  | a struggle to retain a sense of *who we are now*, let alone *who we were*, or *who we are becoming* | DWD133 |
|  |  |  | when I questioned *who will I be* when I die with dementia | DWD158 |
|  |  |  | in this journey towards *my true self* | DWD161 |
|  |  |  | At the centre of our being lies *the true self* … For the person with dementia, this is what remains intact, it is what makes us who we really are | DWD162 |
|  |  |  | I write to remember, to inhabit, for a while, *my earliest self* | MLB45 |
|  |  |  | the disease progressively destroys *the very self that used to be capable* of love and its expression | MLB66 |
|  |  |  | would it be reasonable to expect someone else to pursue my fashion quirks on behalf of *a no-longer-existing Gerda-self*? | MLB208 |
|  |  |  | help me regain *my old self* | LIL35 |
|  |  |  | to touch again *the Diane Friel who had once been*, before she was no more | LIL56 |
|  |  |  | *A different me* clutches the wheel | UTK31 |
|  |  |  | It’s a strange conversation, *the past, present and future versions of ourselves* colliding | UTK34 |
|  |  |  | When did you decide that I was to live a different life *without all the bits that made me me*? | UTK47 |
|  |  |  | They don’t know *the old Wendy* | UTK59 |
|  |  |  | There’s the person I project to those around me, trying to emulate the one they’re more used to, and *the new me* | UTK87 |
|  |  |  | A fuzzy memory that *the old me* would have strolled in confidently | UTK117 |
|  |  |  | I do wake up and wonder, *Which me* am I today? | UTK134 |
|  |  |  | *this new me* | UTK158 |
|  |  |  | *the old me* | UTK196 |
|  |  |  | I know it will become overwhelming and that it *won’t be the me I know there* | UTK230 |
|  |  |  | *a muted version of myself* as I struggle to keep up | UTK230 |
|  |  |  | *You* got the job – of course you did – you were so capable, *so different from me* [She’s talking to herself here.] | UTK239 |
|  |  |  | times when the difference between *the old me and the new me* hits me so hard it leaves me without breath | UTK260 |
|  |  |  | I look back at the WhatsApp conversations. But this one *the old Wendy, the one I knew for fifty-eight years* | UTK261 |
|  |  |  | I am not used to the *two versions of myself* crossing paths, but it had felt that, for a split second, they had met one another | UTK261 |
|  |  |  | As much as I try to adapt to *the person that it has made me* | UTK282 |
|  |  |  | I’m *a different me today* from the one I was six months ago. A different one then from the one I was a year ago | UTK284 |
|  |  |  | Can I rely on *this new me* | UTK284 |
|  |  |  | *the me I knew for fifty-eight years* has already departed | UTK284 |
|  |  |  | *The me now* doesn’t want to go into a care home. But what about *the me that I’ll become*? | UTK285 |
|  |  |  | there was once *another me* for my daughters to call any time of day or night | UTK286 |
|  |  |  | now there’s *this new me* | UTK286 |
|  |  |  | *The positive me* is somewhere else | UTK295 |
|  | a person who’s losing substance | 14 (1.3) | In so many ways, those with Alzheimer’s are like a dandelion—born as a flower, becoming a weed, then *dying from the head down* | OP2 |
|  |  |  | I think of myself now as Mr. Potato Head with *a rotting head* and stick-on body part | OP23 |
|  |  |  | A fish *rots from the head down* | OP32 |
|  |  |  | On bad days, I feel like *a shadow of myself* | OP272 |
|  |  |  | *A cipher* takes your place amid the tubes and tragedy | LMM5 |
|  |  |  | I’m suspended in time, hanging by a *rotting* thread of memory | LMM59 |
|  |  |  | The person in me who lives on until natural death occurs is only *a shadow* left by the deadly laugh of Alzheimer's | LMM117 |
|  |  |  | ending finally as *a near-vegetable rotting* in the sun | LMM181 |
|  |  |  | I write memoir . . . to flesh out *my shrinking self* with former selves | MLB45 |
|  |  |  | *a pale shade* of who I used to be | MLB241 |
|  |  |  | there “was *less of me* every day than there was the day before” | LiL33 |
|  |  |  | I am painfully aware that *less of me* exists than the day before | LiL115 |
|  |  |  | I know there is *an expiration date* on how long I can live independently at home | UTK285 |
|  |  |  | The me I’m left with is *just a shell* | UTK295 |
|  | a person of low status | 10 (0.9) | Sheepishly, like a guilty *young kid*, I assumed the shotgun position | OP167 |
|  |  |  | Perhaps they think I’m *the ‘village idiot’* | OP233 |
|  |  |  | as if I was *a dunce* | OP239 |
|  |  |  | I thought he was going to *fit me with a child harness and leash* | OP271 |
|  |  |  | she ordered sternly, like *a field marshal* | OP281 |
|  |  |  | I often feel I am being treated like *a child* | LMM98 |
|  |  |  | the image of *the fool* is how see myself in the future | DA862 |
|  |  |  | We were expected to withdraw from the world’s stage and be assigned only *the smallest walk-on parts* | DWD53 |
|  |  |  | I doubted his efficiency in that regard, but crossed my fingers. *Beggars* can’t be choosers. | LIL91 |
|  |  |  | *the utterly dependent and exceedingly self-centered bundle of need* that dementia will turn me into | MLB187 |
|  | an animal | 7 (0.6) | I sheepishly shook my head like one of those *cheap toy Chihuahuas* that look out the rear windows of old Chevys | OP274 |
|  |  |  | I *rattle my cage* but no one comes to feed me | LMM185 |
|  |  |  | I rattle my cage but no one comes *to feed me* | LMM185 |
|  |  |  | *a swan* being serene and fairly calm, and presenting in a certain way on the surface, but below the surface paddling sometimes more madly depending on which direction the current is flowing | DA1261 |
|  |  |  | we had become a liability, like *a pet*, a mortgage or yesterday’s laundry | DWD53 |
|  |  |  | This rendered me helpless, stranded with *my tender underbelly*exposed to the vagaries of family and strangers alike | LIL82 |
|  |  |  | toward a treatment of this disease with which we *have been branded* | LIL140 |
| The social experience of dementia is | belonging to a different world | 32 (2.9) | I am *soaked today in exile*, as are others with this disease, as Alzheimer’s ensues | OP253 |
|  |  |  | *one foot on the terra firma we call reality,* *the other south of Eden, a realm beyond the physical* | OP253 |
|  |  |  | I have the feeling sometimes I am *in a motel, an unfamiliar place of transition* | LMM76 |
|  |  |  | I have opened my diaries and journals to you, the reader, as, indeed, I have opened *my world* for you to visit the foggy recesses of my mind | DA225 |
|  |  |  | sensing that *my world* and my freedom within it is shrinking due to my dementia | DA2087 |
|  |  |  | We were expected to *withdraw from the world’s stage* and be assigned only the smallest walk-on parts | DWD53 |
|  |  |  | As dementia survivors, we know both *the world of ‘normals’ and that of dementia* intimately, and we have weathered an extraordinary transition | DWD55 |
|  |  |  | You [The reader] can *enter our reality*, accept more emotion and feeling, and connect with us at this level | DWD141 |
|  |  |  | the stereotype and myths surrounding dementia perpetuate an attitude that *isolates us, into a separate, walled compartment of dementia* | DWD142 |
|  |  |  | *Our world becomes circumscribed* by the stigma of our illness | DWD143 |
|  |  |  | We want to retreat in shame, and do not want *to “come out”* and tell people the diagnosis | DWD143 |
|  |  |  | happening inside our heads. Try to *enter our distorted reality* | DWD148 |
|  |  |  | You need to *enter into our reality*, connect with us by touch, or by look | DWD148 |
|  |  |  | Be imaginative, be creative, try to step across *the divide between our worlds* | DWD148 |
|  |  |  | We know both *your world and ours* | DWD170 |
|  |  |  | We have stepped into *this new world of dementia* | DWD170 |
|  |  |  | It is *as if we are bicultural* and have stepped across the divide between your world and ours | DWD170 |
|  |  |  | It is as if we have stepped across *the divide between your world and ours* | DWD170 |
|  |  |  | *a strange tribe, the Dementers* | MLBix |
|  |  |  | this journal. In it I’ll report my descent into *the post-cerebral realm* for which I am headed | MLB4 |
|  |  |  | Isn’t *letting go of the things of the world*part of the transformation I must undergo as I *enter zombiehood*? | MLB208 |
|  |  |  | I felt I was on a trip to *never-never land* | LIL19 |
|  |  |  | Having always been the outspoken extroverted pivotal center of both my family and group of friends, I was now reluctantly in *the non-contributing purgatory of the early diagnosed* | LIL67 |
|  |  |  | I never expected a publisher to buy it. I didn’t believe the world was ready for *An Alzheimer’s Who Talks*—much less *An Alzheimer’s Who Talks Back*! | LIL121 |
|  |  |  | I didn’t want anyone to speak to me, to *pull me into their world*, because I didn’t know that world, I didn’t know the people in it | UTK48 |
|  |  |  | I didn’t know *that world*, I didn’t know the people in it. *A blank existed between me and them* | UTK48 |
|  |  |  | better to slip *off radar*, however much the guilt and sadness sting | UTK63 |
|  |  |  | Would that make the disease fit better into *the pigeonhole they’ve allocated it?* | UTK259 |
|  |  |  | Twitter brings *the outside world* back in | UTK281 |
|  |  |  | The only thing that remains is the question of when, and that is *the state of limbo I live in* | UTK285 |
|  |  |  | *Limbo* hadn’t arrived then, as we didn’t know what to expect. Now *we live there* | UTK288 |
|  |  |  | When I was first diagnosed we were all *in the unknown* | UTK288 |
|  | being in a bleak place | 19 (1.7) | remarkable *parallels between Alzheimer’s and Pluto*: dense isolation, penetrating silence, a harsh environment, and a world of unthinkable contrasts | OP4 |
|  |  |  | “*Pluto*” .  . . where no one can see you or can hear what is said | OP8 |
|  |  |  | *Pluto* is the perfect place to get lost. Pluto’s orbit is chaotic | OP8 |
|  |  |  | There will come a day when … I won’t return from *this dark, icy place* | OP8 |
|  |  |  | *Pluto’s orbit, like mine at times, is chaotic* | OP33 |
|  |  |  | I am . . . preparing to head for*Pluto, Sedna, and beyond* for the final staging | OP187 |
|  |  |  | the metaphorical analogy between *the dense isolation of Pluto and Alzheimer’s* . . . to understand the isolation of Alzheimer’s and other dementias, just look at the stark photos and illustrations online, black holes and all, an alternate definition of which is “a place where people and things disappear without a trace” | OP193 |
|  |  |  | Rudy knows *the starkness of Pluto* | OP193 |
|  |  |  | then you wander in *a world without certainty and names* | LMM5 |
|  |  |  | Alzheimer’s burns the familiar and turns *the world into an uncertain, frightening place* | LMM78 |
|  |  |  | Alzheimer's sends you back to *an elemental world before time* | LMM89 |
|  |  |  | Alzheimer's sends you back to . . . *a world devoid of possibility and secrets* | LMM89 |
|  |  |  | It is *a world of insecurity*where the certainty of words and the memory of events is unstable | LMM89 |
|  |  |  | It is *a world of abject insecurity and tears of frustration* | LMM89 |
|  |  |  | *the world becomes a freshly unknowable place* where even the simplest things are difficult because they are unrecognizable | LMM89 |
|  |  |  | as the person with dementia struggles to interpret *a world which is now experienced as increasingly chaotic* as dementia progresses | DWD131 |
|  |  |  | trapped by an inability to communicate and to think clearly, to express *this strange mixed-up world being created by our brain damage* | DWD148 |
|  |  |  | *the lonely and scary place of my mother’s dementia—and mine* | MLB127 |
|  |  |  | Dementia can be *a lonely world to live in* | UTK236 |
|  | putting on a pretense | 11 (1.0) | I have *a playbook*, *a script*, backup for everything | OP75 |
|  |  |  | I hope we can throw *the storyline* away, strip *the protective cover*, and tell how we hurt individually from this small, private human catastrophe | LMM146 |
|  |  |  | I am the person behind the *occasionally worn mask of wellness* when the dementia makes more mischief than I can easily hide | DA4943 |
|  |  |  | Often we are just told *the “dementia script”* of decline then death, and given no hope | DWD124 |
|  |  |  | Life had become *an improvisational theater* | LIL64 |
|  |  |  | and I was left *to ad lib* my way through it | LIL64 |
|  |  |  | I continued *playing a camouflage game* of “I’ve Got A Secret” | LIL97 |
|  |  |  | even with Alzheimer’s, life can be fun … if you know how *to play it* | LIL125 |
|  |  |  | I find more ways to cover up the problems, but there are times when I simply can’t *camouflage* the confusion | UTK41 |
|  |  |  | I swallow down the pain that I’m trying *to disguise* with shimmering dust | UTK65 |
|  |  |  | how much longer can I keep up *this facade*? | UTK89 |

**^a^**Full forms of acronyms are given in Table 1. Numbers following acronyms indicate page or location numbers.
